# Genome and cuticular hydrocarbon‐based species delimitation shed light on potential drivers of speciation in a Neotropical ant species complex

**DOI:** 10.1002/ece3.8704

**Published:** 2022-03-10

**Authors:** Rubi N. Meza‐Lázaro, Kenzy I. Peña‐Carrillo, Chantal Poteaux, Maria Cristina Lorenzi, James K. Wetterer, Alejandro Zaldívar‐Riverón

**Affiliations:** ^1^ Colección Nacional de Insectos Instituto de Biología Universidad Nacional Autónoma de México Ciudad de México México; ^2^ Laboratoire d’Ethologie Expérimentale et Comparée UR 4443 LEEC Université Sorbonne Paris Nord Clément France; ^3^ INIFAP Campo Experimental General Terán General Terán Mexico; ^4^ Harriet L. Wilkes Honors College Florida Atlantic University Jupiter Florida USA

**Keywords:** 3RAD, heteroplasmy, neotropics, speciation, ultraconserved elements

## Abstract

Geographic separation that leads to the evolution of reproductive isolation between populations generally is considered the most common form of speciation. However, speciation may also occur in the absence of geographic barriers due to phenotypic and genotypic factors such as chemical cue divergence, mating signal divergence, and mitonuclear conflict. Here, we performed an integrative study based on two genome‐wide techniques (3RAD and ultraconserved elements) coupled with cuticular hydrocarbon (CHC) and mitochondrial (mt) DNA sequence data, to assess the species limits within the *Ectatomma ruidum* species complex, a widespread and conspicuous group of Neotropical ants for which heteroplasmy (i.e., presence of multiple mtDNA variants in an individual) has been recently discovered in some populations from southeast Mexico. Our analyses indicate the existence of at least five distinct species in this complex: two widely distributed across the Neotropics, and three that are restricted to southeast Mexico and that apparently have high levels of heteroplasmy. We found that species boundaries in the complex did not coincide with geographic barriers. We therefore consider possible roles of alternative drivers that may have promoted the observed patterns of speciation, including mitonuclear incompatibility, CHC differentiation, and colony structure. Our study highlights the importance of simultaneously assessing different sources of evidence to disentangle the species limits of taxa with complicated evolutionary histories.

## Graphical abstract

We employed different sources of molecular information in order to assess the species limits of a taxonomically problematic Neotropical ant species complex. We found that species boundaries in this group do not coincide with geographic barriers, and therefore we suggest alternative drivers that may have promoted the observed patterns of speciation, including mitonuclear incompatibility, cuticular hydrocarbon differentiation, and colony structure.

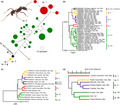

## 1 INTRODUCTION

Species delimitation has become a major task of modern systematics, and the use of different lines of evidence coupled with sophisticated novel methods currently are routinely implemented in studies investigating biodiversity. Multisource approaches that employ different character systems, such as morphological, behavioral, chemical, and DNA sequence data, often help increase rigor for species delineation (Schlick‐Steiner et al., 2010). In particular, genome‐wide DNA data represent a powerful tool not only to resolve disagreements among different data sources by providing robust evolutionary explanations that help to decide among competing hypothesis of species limits (Matute & Sepúlveda, 2019; Quattrini et al., 2019), but also to give detailed insights into the mode and tempo of speciation events (Blair et al., 2018; Feder et al., 2013).

Speciation in sexual organisms is promoted by reproductive isolation between populations (de Queiroz, 2007; Harrison & Larson, 2014; Seehausen et al., 2014). Such reproductive isolation is thought to occur most often between geographically separated populations (Boomsma & Nash, 2014), and allopatric speciation is thus considered the most common mode of species diversification in both animals and plants (Coyne & Orr, 2004; Hernández‐Hernández et al., 2021). However, phylogenetic splits sometimes do not coincide with geographic dispersal barriers (Wollenberg Valero et al., 2019). In such cases, intrinsic factors may help to explain the patterns of geographic distribution and species divergence (Seehausen et al., 2014).

Any process that promotes population divergence could also facilitate speciation (Pfennig et al., 2010). Intrinsic barriers to gene flow, such as genetic incompatibility, genetic drift, ecological selection, and genomic conflict can therefore lead to species divergence (Kulmuni & Westram, 2017; Presgraves, 2010; Seehausen et al., 2014). One proposed mechanism initiated by genomic conflict is the evolution of incompatibilities in co‐functioning mitochondrial (mt) and nuclear genes (Burton & Barreto, 2012; Chou & Leu, 2010; Crespi & Nosil, 2013; Gershoni et al., 2009; Hill, 2015). Co‐evolution of nuclear and mt genomes may result in inter‐population hybrids that display cytoplasmic incompatibilities (Grun, 1976), which create hybridization barriers that may contribute to speciation (Hill, 2015, 2016, 2019). This hypothesis predicts that the mt genotype of each species will be functionally distinct, and that introgression of mt genomes will be prevented by mitonuclear incompatibilities that arise when heterospecific mt and nuclear genes attempt to co‐function to enable aerobic respiration (Hill, 2018, 2019; Sloan, 2015).

At the phenotypic level, communication signals play an important role in species identification, and may contribute to or even drive reproductive isolation and subsequent speciation (Bradbury & Vehrencamp, 1998). In insects, cuticular hydrocarbons (CHCs) play a major role in species recognition, especially in social insects (Blomquist & Bagnères, 2010; Sprenger & Menzel, 2020). CHCs expressed on the insects’ cuticle are essential to prevent desiccation, and have secondarily evolved in communication roles such as nestmate/non‐nestmate discrimination, information about castes, and task specialization (Adams & Tsutsui, 2020; Blomquist & Bagnères, 2010; Chung & Carroll, 2015; van Zweden & d’Ettorre, 2010). As they typically vary among species, CHCs in ants have been used as taxonomic tools to detect morphologically similar, but chemically distinct lineages (Hartke et al., 2019; Lucas et al., 2002; Schlick‐Steiner et al., 2010).

In the present study, we assessed the species boundaries within the *Ectatomma ruidum* species‐complex, a widespread and conspicuous group of Neotropical ants, through integrative analyses of data from 3RAD, ultraconserved elements (UCEs), a fragment of the cytochrome oxidase 1 (*cox1*) mtDNA gene, and CHCs. The ant genus *Ectatomma* Smith, 1858 (Ectatomminae) currently includes 15 valid species with mainly Neotropical distributions (Antweb, 2021). Of these, *E*. *ruidum* (Roger, 1860) is perhaps the most widely distributed. This species was originally described from localities in Brazil, French Guiana, and Colombia (Roger, 1860), though its type locality was subsequently restricted to Colombia (Kugler & Brown, 1982). Currently, *E*. *ruidum* is known to occur from northern Mexico in the state of Tamaulipas to central Brazil, and also on some Caribbean islands (Aguilar‐Velasco et al., 2016). This species inhabits a wide range of environments from sea level to 1600 m of elevation (Kugler & Brown, 1982; Santamaría et al., 2009).

*Ectatomma ruidum* is of particular interest in evolutionary studies due to its taxonomic complexity, wide geographic distribution, and presence of heteroplasmy (occurrence of more than one mtDNA type within an individual) in some of its populations. In the first phylogenetic study carried out for this species‐complex, Aguilar‐Velasco et al. (2016) proposed the existence of four evolutionary lineages plus a presumed hybrid population, based on examination of external morphology and nuclear and mtDNA sequence data. Two of these lineages (*E*. *ruidum* spp. 1 and 2) have broad Neotropical distributions, whereas the others (*E*. *ruidum* spp. 3, 4, and 2 × 3) are apparently restricted to localities along the Pacific coast in southeast Mexico. The authors also reported considerable variation in the mt locus *cox1*, and the existence of nuclear mt paralogs (*numts*; Song et al., 2014) based on the presence of polymorphism in several chromatograms. Meza‐Lázaro et al. (2018) subsequently assembled the mitogenomes of workers assigned to the putative species and the hybrid population proposed in the earlier study using NGS data. The mitogenome assemblies of specimens of some populations from southeast Mexico (*E*. *ruidum* spp. 3, 4 and 2 × 3) had a high number of polymorphic sites, and a detailed examination indicated the presence of two functional, closely related mt genomes within these specimens, one with slow and other with fast evolving haplotypes. These results strongly suggested the presence of extensive heteroplasmy in the latter populations.

Peña‐Carrillo, Poteaux, et al. (2021) analyzed the CHC profiles of specimens assigned to *E*. *ruidum* from populations across its known geographic distribution, focusing on the heteroplasmic populations from southeast Mexico. The CHC profiles varied considerably among populations, supporting the existence of various evolutionary lineages within the complex. More recently, a study of the distress call for members of this complex found that a population in Huaxpaltepec, Oaxaca, Mexico, differs significantly in this trait from other examined populations, strongly suggesting that this population could represent a distinct, undescribed species (Peña‐Carrillo, Lorenzi, et al., 2021).

Here, we performed a comprehensive integrative systematic study based on different sources of molecular evidence to assess the species limits within the *E*. *ruidum* complex. Specifically, we coupled DNA sequence information generated using two reduced genome representation techniques, 3RAD (Bayona‐Vásquez et al., 2019), and UCEs (Faircloth, 2017; Faircloth et al., 2012), with a large data set consisting of a mtDNA sequence fragment of a commonly barcoded locus (Hebert et al., 2003) and CHC profiles, with special attention to heteroplasmic populations from southeast Mexico. Our analyses consistently revealed the existence of at least five distinct species in this ant complex whose species boundaries do not coincide with any known geographic barrier. We therefore discuss the potential role of alternative drivers that may have promoted its species diversification, including mitonuclear incompatibility, CHC differentiation, and colony structure.

## 2 MATERIALS AND METHODS

### 2.1 Taxon sampling

**FIGURE 1:**
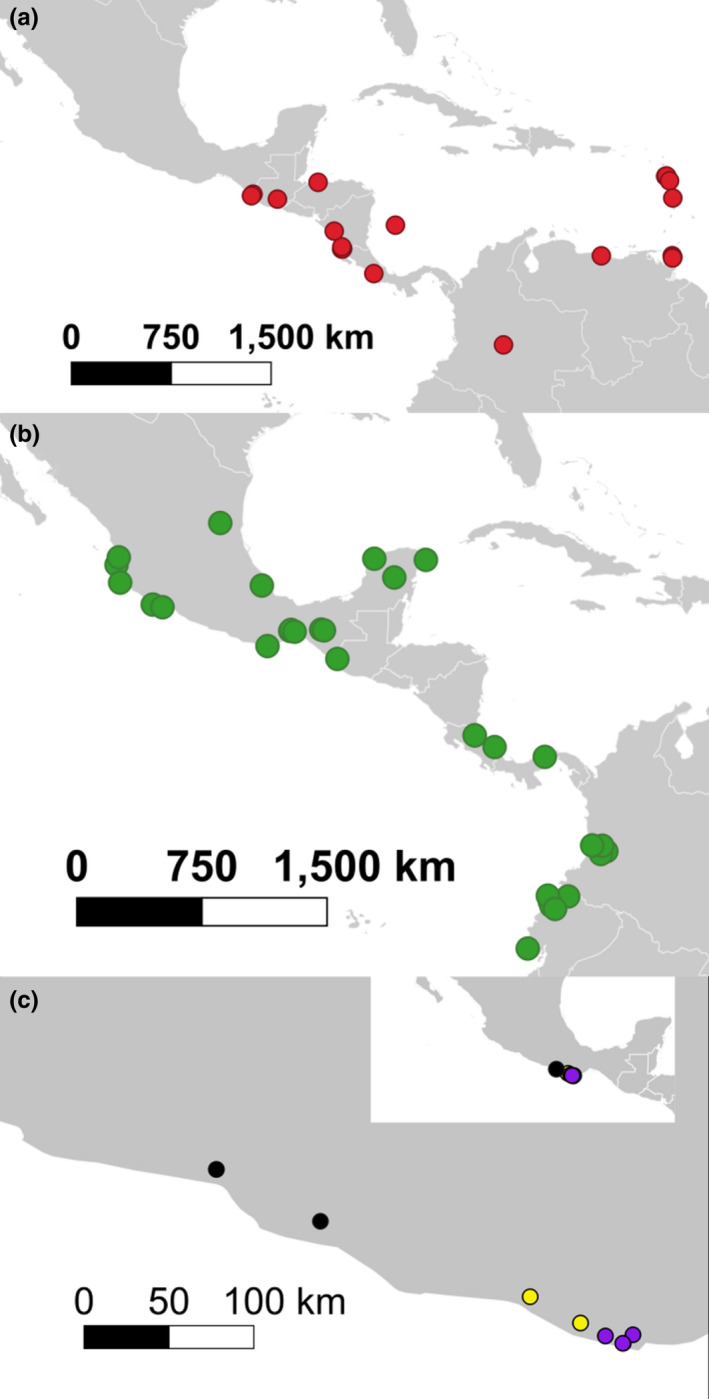
Maps showing the sampled localities for the putative species belonging to the *E*. *ruidum* complex that were delimited in this study: (a) red: *E*. *ruidum* sp. 1; (b) green: *E*. *ruidum* sp. 2; (c) yellow: *E*. *ruidum* sp. 3; violet: *E*. *ruidum* sp. 4; black: *E*. *ruidum* sp. “Pinotepa + Guerrero”

We used worker specimens assigned to *E*. *ruidum* collected from localities across the Neotropics from central Mexico to Colombia, with emphasis on the populations from the state of Oaxaca in southeast Mexico that contain high levels of heteroplasmy (Meza‐Lázaro et al., 2018) (Figure 1). Our sample size varied by location, though in all data sets we included representative specimens of the four putative evolutionary lineages proposed in Aguilar‐Velasco et al. (2016) study (*E*. *ruidum* spp. 1–4 and 2 × 3).

We examined a 626‐bp fragment of the *cox1* gene for 250 specimens assigned to *E*. *ruidum* and one specimen of *E*. *gibbum*, employing the latter as the outgroup. Of these sequences, 107 and 12 were obtained from Aguilar‐Velasco et al. (2016) and Meza‐Lázaro et al. (2018), respectively, whereas 132 were newly generated. We excluded from the data set all potential nuclear mt paralogous sequences (*numts*) that were detected based on their presence of internal stop codons or when they had clearly incorrect phylogenetic relationships (Song et al., 2014). In order to only include orthologous *cox1* sequences, we also detected and excluded all fast‐evolving secondary, paralogous mt copies for the heteroplasmic specimens assigned to *E*. *ruidum* spp. 3, 4, and 2 × 3, employing the phased *cox1* sequences generated in Meza‐Lázaro et al. (2018) as a reference. For the 3RAD and UCE data sets, we generated sequences for 35 (34 *E*. *ruidum*, one *E*. *tuberculatum* as outgroup) and 14 (13 of *E*. *ruidum*, one of *E*. *gibbum* as outgroup) specimens, respectively.

We generated CHC profiles of 24 workers from two localities in Mexico (Puerto Morelos, Quintana Roo, 5 nests; Cuatode, Municipality of Santa María Tonameca, Oaxaca, 6 nests) following the procedure described by Peña‐Carrillo, Lorenzi, et al. (2021), and pooled this information with the data set obtained in the latter study. The complete data set comprised 132 workers and included two to five ants per colony. A list of the specimens used for the different data sets, their taxon assignments, localities information and DNA voucher and GenBank accession numbers is available in the Table S1.

### 2.2 DNA sequencing protocols and assembly procedures

All specimens were preserved in 96% ethanol until processed for DNA sequencing. We extracted genomic DNA from whole specimens using the EZ‐10 Spin Column Genomic DNA Minipreps kit (BIO BASIC^®^, Toronto, Canada) and quantified it using the Qubit fluorometer system (High Sensitivity DNA kit, Life Technologies Inc., Carlsbad, CA, USA). We used 1:10 and 1:30 dilutions of DNA template for Sanger sequencing, and followed the procedures described by Aguilar‐Velasco et al. (2016) for *cox1* amplification and sequencing. Sequences were edited and aligned based on their translated amino acids with the program Geneious version 10.1 (Biomatters, Ltd., Aukland, New Zealand).

We generated genome‐wide sequence data using the 3RAD method (Bayona‐Vásquez et al., 2019). This technique uses three restriction enzymes, two for the construction of dual‐digest libraries and a third that cuts adapter‐dimers formed by the phosphorylated adapter, thus increasing the efficiency of adapter ligation (Bayona‐Vásquez et al., 2019). We digested 250 ng of the extracted genomic DNA for each sample using the XbaI and EcoRI‐HF restriction enzymes (New England Biolabs; Beverly, MA, USA), which leave different sticky ends, and NheI (New England Biolabs; Beverly, MA, USA) to digest iTru adapter dimers. We ligated double‐stranded iTru R1 and iTru R2.1 adapters onto each DNA fragment and ran a short PCR (13–15 cycles) with the iTru5 and iTru7 primers obtained from Adapterama (Bayona‐Vásquez et al., 2019). The resulting libraries were size selected in a 200–800 bp window and sequenced at the Genomic Sequencing Lab facilities at the University of California Berkeley. Libraries were sequenced using the 150 SRR HiSeq2500 Rapid, 10 pM, INDEX (124 M Reads, 72% PhiX Aligned).

We used the process_radtags program implemented in the software pipeline Stacks version 2.0 (Catchen et al., 2011, 2013) to demultiplex, clean, and trim the sequence data. We discarded any read with an uncalled base (‐c) or with low quality scores (‐q). We processed demultiplexed reads using the software pipeline ipyrad version 0.6.19 (Eaton, 2014; Eaton & Overcast, 2020) on the Miztli supercomputer owned by the Dirección General de Cómputo y de Tecnologías de Información y Comunicación, National Autonomous University of Mexico (DGTIC, UNAM). Reads from each sample were clustered using the program VSEARCH version 2.0.3 (https://github.com/torognes/vsearch) and aligned with the program MUSCLE version 3.8.31 (Edgar, 2004).

To avoid the potential for false heterozygous calls due to clustering of paralogs (optimum clustering threshold; Eaton, 2014), we followed the approach described by Ilut et al. (2014) to assess the level of sequence similarity at which two fragments are considered homologous. This approach minimizes the number of false homozygous (a single locus split into two) and false heterozygous (clustering of paralogs) loci in a clustering threshold series. We analyzed a clustering threshold series ranging from 0.80 to 0.98 in 0.02–0.03 increments (0.80, 0.83, 0.85, 0.87, 0.89, 0.91, 0.93, 0.95, and 0.98). We also conducted maximum likelihood (ML) phylogenetic analyses as described below for the matrices with the above clustering threshold values to evaluate their level of nodal support. Based on the results obtained from these two approaches, we selected a clustering threshold value of 0.98 to build four matrices with min_sample_locus values of 25, 28, 30, and 33 including the outgroup and one with a min_sample_locus of 33 excluding it.

We generated UCE data from libraries following Branstetter et al. (2017), and included previously generated UCE data from four male specimens (Meza‐Lázaro et al., 2018). We fragmented up to 50 ng of input DNA to an average fragment distribution of 400–600 bp using a Qsonica Q800R (Qsonica LLC, Newton, CT) or a BioRuptor^®^ Pico sonicator (Diagenode, Liége, Belgium). Following DNA fragmentation, we constructed sequencing libraries using the Kapa library preparation kit (Kapa Biosystems Inc., Wilmington, MA) and custom dual‐indexing barcodes (Glenn et al., 2019). We purified PCR reactions 0.8–1.0× using Sera‐Mag™ SpeedBeads (Thermo‐Scientific, Waltham, MA, USA) (Rohland & Reich, 2012).

We pooled 10–12 libraries at equimolar concentrations for UCE enrichment, adjusting pool concentrations to 147 ng/μl. We used a total of 500 ng of DNA (3.4 μl each pool) for each enrichment. We enriched each pool using the bait set “ant‐specific hym‐v2” (Branstetter et al., 2017), which has 9446 custom‐designed probes (MyBaits, MYcroarray, Inc., Ann Arbor, MI, USA) targeting 2524 UCE loci and 452 baits targeting 16 commonly sequenced exons. The enriched library quality was verified using an Agilent TapeStation 2200 (Agilent Tech, Santa Clara, CA, USA). We sent pools to the University of Utah Genomics Core facility and to the Georgia Genomics Facility at the University of Georgia, where they were sequenced on an Illumina HiSeq 2500 (PE150) and an Illumina HiSeq X Ten (PE150), respectively.

Raw data were demultiplexed and converted from BCl to FASTQ by the sequencing facilities. We used the software package PHYLUCE version 1.5.0 and its associated programs (Faircloth, 2016) for assembly and alignment of the UCE data. We cleaned and trimmed raw reads using ILLUMIPROCESSOR (Faircloth, 2013). The cleaned and trimmed reads were assembled de novo using the program ABySS version 1.3.6 (Simpson et al., 2009). We mapped the assembled contigs to the hym‐v2 bait database to identify individual UCE loci, to remove paralogs and to generate a list of shared UCE loci. We sorted out data by locus and aligned each one with the program MAFFT version 7.130b (Katoh et al., 2019). The resulting alignments were filtered and trimmed with the program Gblocks version 0.91b (Castresana, 2000; Talavera & Castresana, 2007). We analyzed matrices with 75, 80, 90, 95, and 100% taxon occupancy (percent of taxa required to be present at each locus).

We followed the Tutorial II: Phasing UCE data (Faircloth, 2021) to call for SNPs, which is derived from the procedure described by Andermann et al. (2019). The above workflow requires an individual‐specific “reference” that can be aligned against raw reads. Hymenopteran males are haploids, and thus we only expected homozygous loci for them. We used edge‐trimmed exploded alignments as reference contigs and aligned raw reads to them. We exploded the edge trimmed alignments to create separate FASTA files for each sample using phyluce_align_explode_alignments. We used BWA‐MEM to map the FASTQ read files against the contig reference database for each sample. We sorted the reads within each bam file into two separate bam files using phyluce_snp_phase_uces. We built three final matrices based on filtering UCE loci with 85%, 90%, and 100% taxon occupancy. We also used the exploded alignment and raw reads of the 100% taxon occupancy matrix to build an additional matrix phasing the data and calling a single variant SNP per locus.

### 2.3 Phylogenetic analyses

#### 2.3.1 Cox1

We conducted a ML phylogenetic analysis for the *cox1* data set using the program RAxML version 8 (Stamatakis, 2014) with bootstrap replicates. We considered three partitions according to codon positions and used the GTR + Γ model of sequence evolution for each of them. Branches with bootstrap support (BTP) ≥70% were considered as well supported. We also built a haplotype network in POPART (Leigh & Bryant, 2015) using TCS (Clement et al., 2002) to have a better visualization of the relationships among *cox1* haplotypes.

#### 2.3.2 3RAD

We carried out ML and Bayesian phylogenetic analyses with the four selected matrices including the outgroup (clustering threshold = 98%, min sample locus= 25, 28, 30, 33). The ML analyses were conducted with the program RAxML version 8.0 (Stamatakis, 2014) using the GTR‐GAMMA model. Branch support was estimated using the automatic bootstrap function, which calculates a stopping rule to determine when enough replicates have been generated (Pattengale et al., 2010). We conducted the Bayesian analyses with the program ExaBayes version 1.5 (Aberer et al., 2014). These analyses were run using the generalized time‐reversible model (GTR + G) and five independent MCMC chains of 1,000,000 generations each. The first 100,000 trees (10%) were discarded as burn‐in for each MCMC run prior convergence (i.e., when maximum discrepancies across chains <0.1). We assessed burn‐in, convergence among runs and run performance examining the resulting parameter files with the program TRACER version 1.7.0 (Rambaut et al., 2018). We computed consensus trees using the consensus utility of ExaBayes.

We also employed a coalescent‐based species tree estimation method for the 3RAD data sets. The RADseq techniques generate relatively short sequences from a large number of loci, where the potential informative gene tree variation within a single RAD locus is small (Eaton & Ree, 2013). For the 3RAD data we therefore used the program SVDquartets version 1.0 (Chifman & Kubatko, 2014) implemented in PAUP version 4.0a (Swofford, 2003), since it allows the use of multi‐locus or SNP data under the coalescent model bypassing gene tree reconstruction (Chou & Leu, 2015). We assessed variability in the estimated tree using a nonparametric bootstrapping with 500 replicates.

#### 2.3.3 UCEs

We conducted ML analyses for the three UCE matrices (90%, 95%, 100% taxon occupancy) using the program RAxML version 8 (Stamatakis, 2014) with the best tree plus rapid bootstrap search (“‐f a” option) and 200 bootstrap replicates. We used the GTR + Γ model of sequence evolution for the best tree and bootstrap searches. We carried out these analyses using three different partition schemes (unpartitioned, data partitioned by locus, data pre‐partitioned by locus). We selected the best evolutionary model for these partitions using the program PARTITIONFINDER version 2 (Lanfear et al., 2017) based on the Bayesian Information Criterion and the rcluster option, which is more appropriate for larger data sets.

We carried out Bayesian analyses with the program Exabayes version 1.4.1 (Aberer et al., 2014). Each analysis consisted of two independent runs of 10 million generations each, two independent runs with Metropolis–Coupling in parallel to better sample parameter space, three heated and one cold chain per run, and sampling trees every 1000 generations. We linked branch lengths across partitions and ran each partitioned search for one million generations. Mixing and stationarity were monitored with the program TRACER version 1.7.0 (Rambaut et al., 2018). We built consensus trees using the consensus utility contained in the program Exabayes version 1.4.1 (Aberer et al., 2014) using a burn‐in of 25%. To evaluate for the presence of reticulation, we used the neighbor‐net method (Bryant & Moulton, 2004) implemented in the program SplitsTree (Huson & Bryant, 2006) for computing an unrooted phylogenetic network based on the alignment of the concatenated phased loci with 100% taxon occupancy.

For tree estimation with the UCE data based in the multispecies coalescent model, we employed the program Astral v.4.10.8 (Mirarab et al., 2014; Mirarab & Warnow, 2015), a coalescent‐based method that is based on gene tree reconstruction. For this analysis, we first generated gene trees from the 100% taxon occupancy matrix, phasing loci with the program RaxML with 200 bootstrap replicates. We then used the resulting gene trees to carry out a subsequent analysis with ASTRAL, using unrooted trees and missing data. We calculated nodal support with 200 multi‐locus bootstrap replicates (Seo, 2008).

### 2.4 Genetic structure and species delineation analyses

We employed the program STRUCTURE version 2.3.4 (Pritchard et al., 2000) implemented in the ipyrad.analysis toolkit (https://ipyrad.readthedocs.io/analysis.html), to assess patterns of genetic structure and admixture among the examined populations with the 3RAD data. We used the 98_33 matrix (clustering threshold = 98 and min_sample_locus = 33, outgroup excluded) to perform an individual‐based Bayesian clustering analysis. We used an admixture model with correlated frequencies and assessed values of population differentiation (*K*) in 15 independent runs for each *K* from 2 to 8. All runs were conducted with the first 250,000 being discarded as burn‐in. Figures were generated based on the iterations with the highest posterior probability. The optimal *K* value was determined based on the highest average likelihood value [LnP(D)] obtained (Evanno et al., 2005). However, we also reported the different model solutions across *k* values that we explored in order to have a broader understanding of the organization of genetic variation and potential admixture in the examined populations (Driscoe et al., 2019).

We carried out species delimitation analyses with the 3RAD data using the program Bayesian Phylogenetics & Phylogeography version 3.3 (BPP; Flouri et al., 2018; Yang & Rannala, 2010, 2014). BPP evaluates speciation models using a reversal jump Markov Chain Monte Carlo (rjMCMC) algorithm to determine whether to collapse or retain nodes in the phylogeny, assuming no admixture following a speciation event (Yang & Rannala, 2010). BPP requires an input guide tree representing the species phylogeny with all possible species (Leaché & Fujita, 2010). We used the topology derived from the analysis with the 98_33 matrix as guide tree and considered six putative species: Aguilar‐Velasco et al.’s (2016) *E*. *ruidum* spp. 1, 3, 4, the putative hybrid 2 × 3 (considering samples from Guerrero and Oaxaca), and *E*. *ruidum* sp. 2 split into two putative species according to the geographically congruent clades that were recovered in the phylogenetic analyses with the UCE and 3RAD data.

We randomly subsampled the 98_33 matrix to produce three different matrices with 200 loci and 10 with 50 loci. We examined three sets of parameters varying the ancestral population size (*θ*) and root age (*τ*). The first assumed large ancestral population sizes and deep divergences [θ G (1, 10) and τ0G(1, 10)], the second small ancestral population sizes and shallow divergences among species [θ G(2, 2000) and τ0G(2, 2000) 3], and the third large ancestral populations sizes and relatively shallow divergences among species [θ G(1, 10) τ0 G(2, 2000)]. We ran analyses for all models without data to separately evaluate the effects of the data and parameters. We subsequently ran seven replicates for one of the matrices with 50 loci with each set of parameters to check whether the analyses were run long enough, and then ran analyses for all matrices with five replicates each starting from different seeds. A significant posterior probability (PP ≥0.95) value was employed across all runs to retain a given node (i.e., indicating lineage splitting). All analyses were run for 500,000 generations (first 10,000 were burn‐in), with a sampling interval of 50.

We conducted a Bayes Factor species delimitation (BFD) analysis (Grummer et al., 2014; Leaché et al., 2014) for the phased UCE 100% taxon occupancy matrix calling a single variant SNP per locus. The BFD approach compares candidate species delimitation models with different numbers of species, estimating the marginal likelihood of each competing species delimitation model, ranking models by marginal likelihood and estimating Bayes factors to assess support for model rankings (Kass & Raftery, 1995).

We conducted a Bayes factor delimitation (BDF) analysis following Leaché and Bouckaert’s (2018) tutorial. This tutorial implements SNAPP (Bryant et al., 2012), available in the BEAST2 platform (Bouckaert et al., 2014). SNAPP bypasses the necessity of having to explicitly integrate or sample gene trees at each locus, and it codes SNP data as follows: individual homozygous for the original state = “0,” heterozygous = “1,” homozygous for derived state = “2.” We used a python function to extract biallelic SNPs directly from allele Multiple Sequence Alignments (snps_from_uce_alignments.py, available from: github.com/tobiashofmann88/snp_extraction_from_alignments/; Andermann et al., 2019). We compared species delimitation models differing in the number of species. The base model had five species (*E*. *ruidum* spp. 1–4, 2 × 3), whereas the alternative ones were collapsed into one, two, three, and four species in different combinations. The path sampling parameters, which are used to estimate marginal likelihood, were set to 12 and 24 steps with chainLength = 1,000,000.

All the analyses conducted using the 3RAD and UCE data sets were run on the CIPRES Web Portal (Miller et al., 2010) or on the Miztli supercomputer owned by the Dirección General de Cómputo y de Tecnologías de Información y Comunicación, Universidad Nacional Autónoma de México (DGTIC, UNAM).

### 2.5 CHC analyses and genetic distances

We calculated uncorrected genetic distances for the 3RAD (98_33 matrix), UCEs, and *cox1* data sets. *Cox1* distance matrices were calculated using average distance per population, whereas for the 3RAD data set distances were calculated only using SNPs. All genetic distances were calculated using the program Mega X (Kumar et al., 2018).

We used the Bray–Curtis distances as dissimilarity measures to calculate chemical distances between the 156 examined specimens based on the relative abundances of the simplified chemical profile. This was obtained by summing the percentages of hydrocarbons with the same carbon‐chain length by class (Peña‐Carrillo, Poteaux, et al., 2021). We then calculated the chemical distance between populations (centroids) and used them to obtain a hierarchical cluster dendrogram using the program Primer 6 & Permanova + (Anderson, 2017). We performed Mantel tests based on Pearson's product–moment correlation, running analyses with a maximum of 999 permutations to correlate chemical and genetic (*cox1*, 3RAD and UCEs) with geographic distances among the examined populations. Mantel tests were performed with the Vegan package version 2.5.7 (Oksanen et al., 2016) implemented in R Studio version 3.5.2 (RStudio Team, 2021).

## 3 RESULTS

### 3.1 Genome‐wide data

The samples included in the 3RAD data set had from 75,193 to 1,007,069 reads, and the generated matrices contained 986–7094 loci (Table S2). The outgroup employed for this data set, *E*. *tuberculatum*, shared between 375 and 1322 loci with the ingroup. The matrices derived from the unphased UCE data varied from 642 to 2196 loci and from 508,859 to 1,817,455 characters (100% and 75% taxon occupancy matrices, respectively; Table S3). The phased UCE matrices had considerably fewer loci than those with unphased data (737 and 390 loci for the 85% and 100% taxon occupancy matrices, respectively), though the unphased data contained more parsimoniously informative sites (Table S3). SNP codification as a binary matrix for the UCE data showed the presence of heterozygous loci in the male samples, which are also present in the nucleotide alignments.

### 3.2 Phylogenetic analyses

The ML phylogram derived from the *cox1* data set (Figure S1) recovered the putative species *E*. *ruidum* sp. 1 and *E*. *ruidum* sp. 2 each as monophyletic (BTP = 98 and 60, respectively). The specimens assigned to *E*. *ruidum* spp. 3–4 and 2x3 on the other hand appeared intermingled in a single clade (BTP = 76). *Ectatomma ruidum* sp. 1 was sister to the remaining taxa, but with low support (BTP = 0.27). Most of the internal relationships within *E*. *ruidum* sp. 1, formed by specimens from southern Mexico, Guatemala, Honduras, Nicaragua, Costa Rica, Colombia, Venezuela, and the Lesser Antilles, were unresolved. In contrast, *E*. *ruidum* sp. 2 had geographic structure, being composed of four main subclades with unresolved relationships among them. One of these clades contained specimens from Ecuador (BTP = 92), a second specimens from the remaining South and Central American localities (BTP = 54), a third specimens from Quintana Roo in southeast Mexico (BTP = 76), and the fourth specimens from southeast, central, and northeast Mexico (BTP = 10).

**FIGURE 2:**
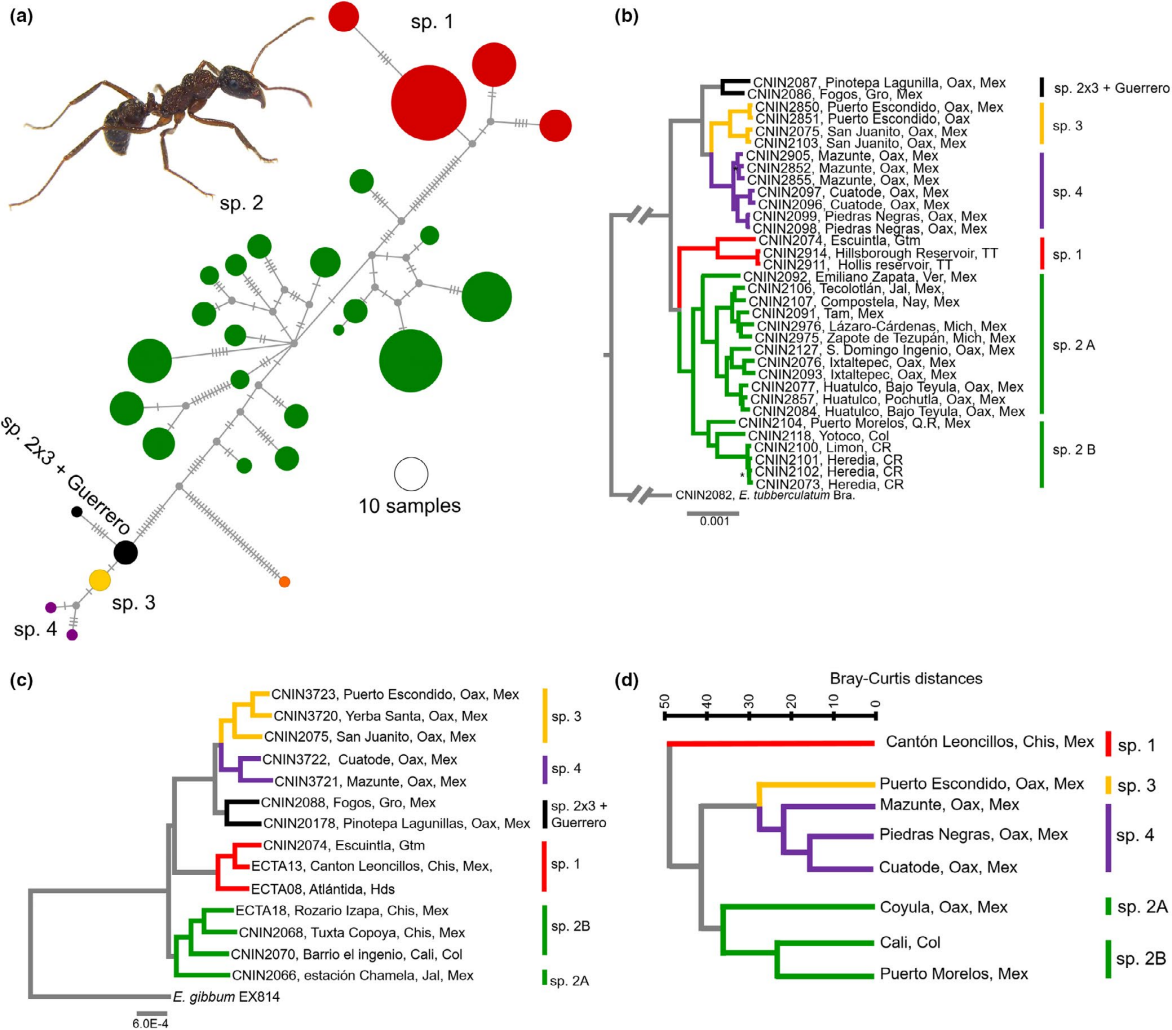
Summary of the results obtained with the different sources of information examined in this study: (a) hierarchical cluster dendrogram built with the data set of the simplified CHCs profile of the *E*. *ruidum* species complex; (b) Bayesian phylogram derived from the 3RAD data set (minimum sample locus = 30); (c) ML phylogram derived from the UCEs data set (95% taxon occupancy matrix); (d) haplotype network reconstructed with the *cox1*data set. Photograph: *E*. *ruidum* sp. 2, Cali, Colombia

The *cox1* haplotype network showed that the *E*. *ruidum* haplotypes are grouped into three main haplogroups, which are separated from each other by 17–20 mutational steps (Figure 2a). Two of these haplogroups are each represented by specimens assigned to *E*. *ruidum* spp. 1 and 2, whereas the remaining one had members of *E*. *ruidum* spp. 3, 4, and 2 × 3. Moreover, the haplogroup with specimens of *E*. *ruidum* sp. 2 was divided into various geographically structured clusters.

The phylogenetic analyses with the 3RAD data set yielded well resolved, highly supported topologies. All the topologies derived from the ML, Exabayes and SVDquartets’ analyses recovered four main clades with significant support (Figures 2b and S2). One of these clades contained members of *E*. *ruidum* sp. 1, which was sister to a second clade represented by the specimens of *E*. *ruidum* sp. 2. These two taxa were sister to the two remaining main clades, one having four of the five specimens of *E*. *ruidum* sp. 3 and the two specimens of *E*. *ruidum* sp. 4, and the other the specimen assigned to *E*. *ruidum* sp. 2 × 3 from a locality near Pinotepa Nacional, Oaxaca, and the specimen of *E*. *ruidum* sp. 3 from Guerrero, Mexico. The main clade formed by samples assigned to *E*. *ruidum* sp. 2 was further divided into two subclades. One was exclusively composed of specimens from southeast (Yucatan and Chiapas) and northeast (Tamaulipas) Mexico (*E*. *ruidum* sp. 2A), and the second comprised specimens from Central America, including Quintana Roo in southeast Mexico, and South America (*E*. *ruidum* sp. 2B).

Most of the UCE analyses carried out with the ML, Exabayes and species tree methods recovered the above four clades with the same relationships among them mostly with strong support (Figures 2c and S2). However, in contrast to most of the 3RAD topologies, the ML phylograms based on the 90, 95, and 100% occupancy matrices recovered *E*. *ruidum* sp. 1 as sister to the clade formed by *E*. *ruidum* sp. 3, 4, and 2x3 (BTP = 100).

### 3.3 Genetic structure

**FIGURE 3:**
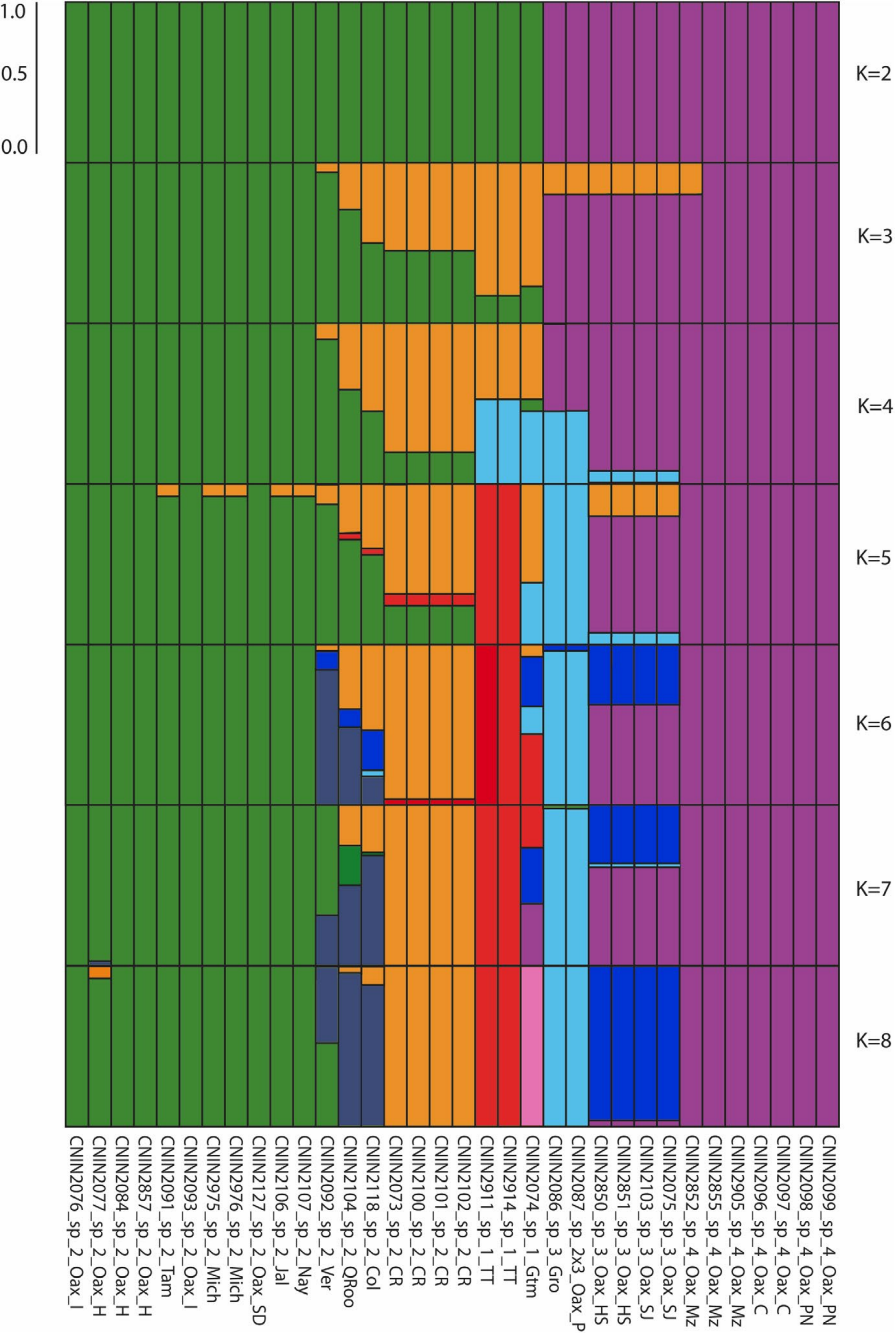
Patterns of genetic structure and admixture among the examined populations based on the 3RAD data set (98_33 matrix) for values of population differentiation (*K*) from 2 to 8

The STRUCTURE analyses carried out with the 3RAD data set displayed the highest value of Ln P(D) at *K* = 7, though the Evanno method had its highest value at *K* = 3 (Figure 3). At *K*= 2, we recovered one cluster with members of E. *ruidum* sp. 1 and E. *ruidum* sp. 2 and another with those of *E*. *ruidum* spp. 3, 4, and 2 × 3. At *K*= 3 the specimens of *E*. *ruidum* sp. 1 from Guatemala and Trinidad and Tobago formed a relatively well‐differentiated cluster. At subsequent *K* values, the specimens from Pinotepa and localities in Guerrero in Mexico, and the ones from Trinidad and Tobago were each recovered as exclusive clusters, whereas at *K* = 7 and 8 the specimens representing *E*. *ruidum* sp. 3 (except the one from Guerrero), *E*. *ruidum* sp. 4, *E*. *ruidum* sp. 2 from Quintana Roo, Mexico and Colombia, and the one from Guatemala each formed well‐differentiated clusters.

### 3.4 Species delineation

The BPP analyses conducted with 3RAD significantly supported the species model that considered six evolutionary lineages in all replicates (PP > 0.95; Table S4), the four putative species *E*. *ruidum* spp. 1, 3, and 4, the specimens from Pinotepa (Oaxaca) and Guerrero regarded as *E*. *ruidum* sp. 2 × 3, and the two groups of *E*. *ruidum* sp. 2 (*E*. *ruidum* sp. 2A, 2B). The only collapsed lineages found in some replicates were for the split of *E*. *ruidum* spp. 3 and 4 into more than two species, thus supporting the existence of only two separate lineages for these populations.

The BFD comparisons of the two tested species delimitation models using the phased UCE data consistently favored a five species model, with *E*. *ruidum* spp. 1–4, as well as the specimens from Pinotepa (Oaxaca) and Guerrero (*E*. *ruidum* sp. 2 × 3), Mexico, each representing separate species (Table S5).

### 3.5 CHC‐based distances

The cluster analysis carried out on the CHCs yielded two main clusters (Figure 2d). One belonged to the hydrocarbon profiles of *E*. *ruidum* sp. 1 and is clearly separated from the second, which contained two subclusters, one with the *E*. *ruidum* sp. 2 samples and the second with specimens assigned to *E*. *ruidum* spp. 3 and 4. The *E*. *ruidum* sp. 2 cluster showed that the CHC profiles of the populations from Colombia and Puerto Morelos, in southeast Mexico, were more similar to each other than those of the population from Coyula, Oaxaca, Mexico (Figure 2d). We also found that the CHC profiles of E. *ruidum* sp. 3 were different from those of the populations of *E*. *ruidum* sp. 4.

**FIGURE 4:**
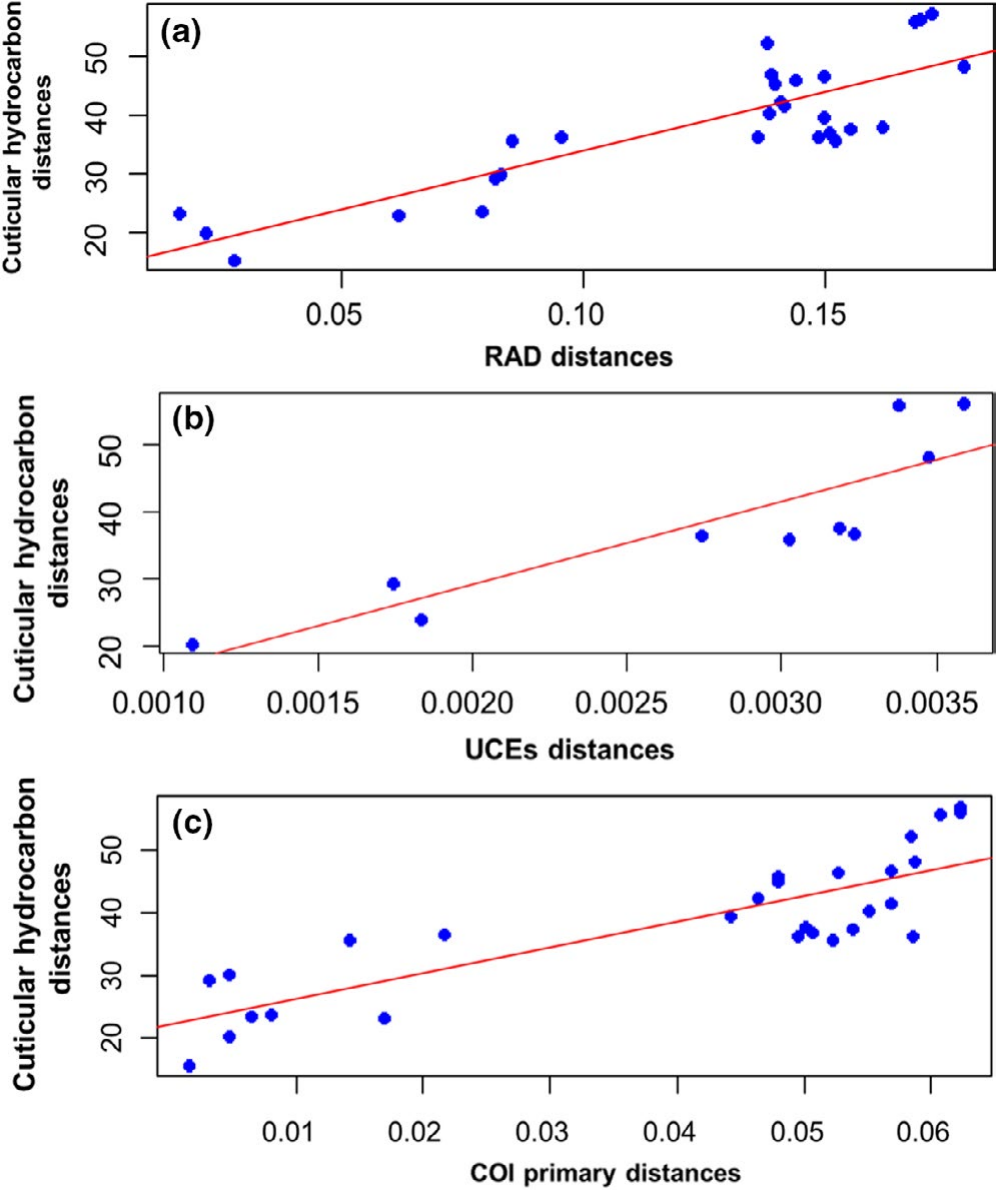
Pearson's product‐moment correlation between CHC and 3RAD, UCEs, and *cox1* distances

Chemical distances were significantly correlated with the genetic distances obtained for the 3RAD (Mantel test, *r* = 0.855, *p* = .001), UCE (*r* = .876, *p* = .016), and *cox1* (*r* = .847, *p* = .004) data sets (Table S6; Figure 4). In contrast, there was no significant correlation between geographic and chemical distances (*r* = −.022, *p* = .354), nor between geographic and genetic distances calculated from 3RAD (*r* = 0.322, *p* = .141), UCEs (*r* = .052, *p* = .225), *cox1* primary haplotypes (r = 0.217, *p* = .205) and *cox1* secondary haplotypes (r = −.258, *p* = .826) (Table S6).

## 4 DISCUSSION

We used simultaneous assessment of nuclear and mtDNA along with CHC data to disentangle the species limits in a morphologically conserved insect taxon with an intricate evolutionary history. Morphological and genetic (mainly mt) evidence gathered in previous studies suggested the probable existence of four distinct evolutionary lineages within the *E*. *ruidum* complex (Aguilar‐Velasco et al., 2016; Meza‐Lázaro et al., 2018). However, those studies were inconclusive due to the presence of mt heteroplasmy in individuals of some populations from Oaxaca, which led to the reconstruction of clades with considerably long branches due to the preferential sequencing of the mt haplotype with the faster substitution rate over the alternative haplotype from the same specimen. Moreover, the mt markers recovered highly genetically structured populations, which is frequent in social hymenopterans due to extreme female philopatry (Hakala et al., 2019; Johnstone et al., 2012), whereas the nuclear markers had almost no variation. Below we discuss our results from two different nuclear genomic sequence data, which, together with the mt and CHC information, overcome limitations of the previous works, providing a robust framework of species delimitation that can be employed for further taxonomic and evolutionary studies in the group.

### 4.1 Integrative species delimitation

The analysis of independent lines of evidence often yields conflicting results of species delineation because distinct secondary properties (e.g., morphological and genetic distinctiveness, monophyly, reproductive isolation) can be acquired by lineages in different times during the course of divergence (de Queiroz, 2007). Integrating molecular and phenotypic data using different methodologies is therefore widely assumed to be the most effective approach for delimiting species (Dayrat, 2005; Pante et al., 2015; Schlick‐Steiner et al., 2010; Will et al., 2005). Integrative taxonomy provides statistical rigor for species delineation and validation of species, as well as for assignment of specimens to a given species group, improving the detection of cryptic diversity and the inference of relationships among species (Edwards & Knowles, 2014; Leaché & Fujita, 2010; Leavitt et al., 2015; Schlick‐Steiner et al., 2010). This approach allows the identification of concordant patterns of divergence based on different sources of information (e.g., unlinked genetic loci, morphology, behavior), thus revealing cases of full lineage separation, since it is highly unlikely that a coherent pattern of character agreement emerges by chance (Padial et al., 2010).

Our molecular‐based analyses using the *cox1*, 3RAD, and UCEs data sets, together with analysis of CHC variation, yielded strongly congruent evidence for the existence of five different evolutionary lineages among the examined populations of *E*. *ruidum*. Our results support the existence of the four species suggested by Aguilar‐Velasco et al. (2016) (*E*. *ruidum* spp. 1–4) and also a fifth species corresponding to what they suggested to be a hybrid population (*E*. *ruidum* 2 × 3). Four of these species were found in localities situated along the lowlands of the Sierra Madre del Sur in Oaxaca and Guerrero, Mexico. Moreover, the BPP analyses with 3RAD and the CHC evidence divided *E*. *ruidum* sp. 2 into two separate species, one containing specimens from Colombia to southeast Mexico in Quintana Roo, and the other specimens from various localities from southeast to central and eastern Mexico. However, the UCE and *cox1* data sets did not show strong evidence to confirm this, and thus additional data are needed to corroborate whether the above taxon actually consists of two evolutionary lineages. Also, the possibility of an additional species in the Pacific coast of Oaxaca, Mexico, is plausible according to recent chemical, genetic, and acoustic studies (Peña‐Carrillo, Poteaux, et al., 2021).

We consistently recovered the specimens assigned to *E*. *ruidum* sp. 1 as a well‐differentiated species, regardless of the data set and analyses employed. Our CHC and *cox1*‐based network showed the members of *E*. *ruidum* sp. 1 as a highly divergent cluster whose geographic distribution ranges from southeast Mexico to Ecuador, Colombia and Venezuela in South America, and in the Lesser Antilles in the Caribbean. This network also showed the existence of geographic structure within this species. The name *E*. *ruidum* should be applied to the populations assigned to *E*. *ruidum* sp. 1 based on the type locality of the species, which was restricted to Colombia by Kugler and Brown (1982), and on their morphological correspondence with the syntypes from this country (Aguilar‐Velasco et al., 2016). The 3RAD, UCE, and *cox1* analyses also recovered the specimens of *E*. *ruidum* sp. 2 as a separate species, being composed of two geographically structured clades. One of these clades included specimens from southeast Mexico to Colombia and Ecuador in South America, whereas the other one was represented by specimens from southeast to northern Mexico. The *cox1* data set, however, showed a considerable genetic distance between these clusters, suggesting that they could be separate species. This hypothesis is supported by the CHC analyses, which also showed a marked divergence between the two clades.

The remaining three species delimited here correspond to populations from the lowlands of Sierra Madre del Sur in the states of Oaxaca and Guerrero, in southeast Mexico. The two genome‐wide data sets and the CHC profiles indicated that *E*. *ruidum* sp. 3 and *E*. *ruidum* sp. 4 are two closely related species whose geographic distribution is restricted to lowland areas of Oaxaca. Moreover, our results consistently support the hypothesis that the specimens of the putative hybrid population proposed by Aguilar‐Velasco et al. (2016) from Pinotepa Nacional, Oaxaca, and those assigned to *E*. *ruidum* sp. 3 from the state of Guerrero, actually represent a distinct species. Further comprehensive sampling on foothills along the Pacific coast in southeast Mexico will reveal the actual geographic distribution of these three species. Moreover, taxonomic inferences based on these results should consider the previous name availability within the group. *Ectatomma aztecum* was described by Emery (1901) based on a single specimen collected in the state of Michoacán, Mexico, but without precise locality; however, it was subsequently regarded as a synonym of *E*. *ruidum* by Kugler and Brown (1982). According to Aguilar‐Velasco et al. (2016) the syntype of *E*. *aztecum* (CASENT0903841; MSNG, Genoa, Italy) is morphologically similar to the specimens assigned to *E*. *ruidum* sp. 3.

### 4.2 Potential drivers of speciation in the *E. ruidum* complex

Allopatric speciation, which involves geographic isolation of segments of a formerly contiguous population, is firmly established as the primary mechanism by which new species evolve (Coyne & Orr, 2004; Hernández‐Hernández et al., 2021). Without spatial isolation among populations, it is difficult to draw a scenario in which gene flow can be reduced or eliminated. However, it is known that speciation can be multifactorial with multiple axes of differentiation characterizing even incipient species (Brodetzki et al., 2019; Mullen & Shaw, 2014). For instance, in *Heliconius* butterflies color pattern divergence, mate preference, host‐plant use, and microhabitat choice possibly are implicated in species diversification (Brown, 1981; Estrada & Jiggins, 2002; Mallet et al., 2007; Smiley, 1978). In the case of the species of the *E*. *ruidum* complex, we do not see any clear correlation between their distribution pattern and geographic barriers, nor possible events of microallopatry or niche specialization. For instance, in the state of Chiapas, Mexico, the populations of *E*. *ruidum* sp. 1 and sp. 2 occurred in quasi‐sympatry, at <15 km from each other (Lachaud, 1990). Even more puzzling is the geographic distribution of the three delimited species restricted to lowland areas of Oaxaca and Guerrero, in southeast Mexico, which we found to be separated from each other only by 5–30 km. Below we discuss three potential phenotypic and genotypic factors that could have promoted the speciation among the species of the *E*. *ruidum* complex: mitonuclear conflict, chemical cue divergence, and colony structure.

Mt performance affects every aspect of individual fitness and its correct function relies on mitonuclear compatibility (Angers et al., 2018; Zaidi & Makova, 2019). This intimate interaction between the mt and nuclear products has led to a strong coevolution between both genomes (Blier et al., 2001; Burton et al., 2013; Chou & Leu, 2015). It has been proposed that mitonuclear coevolution in isolated populations triggers speciation, since population‐specific mitonuclear coadaptations lead to between‐population mitonuclear incompatibility, thus precluding gene flow (Hill, 2016). It is therefore plausible that the rapidly evolving mt haplotypes found in the three heteroplasmic species of the *E*. *ruidum* complex restricted to southeast Mexico played a role in their speciation if they were expressed and interacted with the nuclear products, consequently pressing for its evolution (Hill, 2017). This could have been achieved through a marked population structure promoted by assortative mating, which created mitonuclear incompatibilities and barriers to gene flow between the taxa involved. However, since heteroplasmy does not seem to occur in the widely distributed *E*. *ruidum* spp. 1 and 2, other factors probably could also have promoted the species diversification in this complex.

Social behavior and recognition cues could act as major traits that could drive assortative mating, limit gene flow and ultimately promote speciation (Hochberg et al., 2003). Sympatric speciation based on social trait divergence has been suggested for ants of the *Cataglyphis niger* complex, where three incipient species possess consistent differences in CHC composition, social structure and mtDNA sequence data (Brodetzki et al., 2019). Our results showed that the CHC distances among populations mirrored their phylogenetic relationships obtained from mt and genome‐wide data. A similarly high correlation between CHC and genetic data has been observed in other social insects (Isoptera: Dronnet et al., 2006; stingless bees: Leonhardt et al., 2013; ants: Hartke et al., 2019). CHC divergence has been associated with nestmate/non‐nestmate communication (van Zweden & d’Ettorre, 2010) and may also play an important role in pre‐mating isolation (Savarit et al., 1999; Smadja & Butlin, 2009; Snellings et al., 2018).

Another important trait in social insects is colony structure. In some ant groups, shifts from monogynous to polygynous colonies and gyne morphological polymorphism (macro and microgynes) are also known to be involved in their speciation (Brodetzki et al., 2019; Seifert, 2010). For instance, limited dispersal of females of polygynous species can promote differentiation between populations, and if male dispersal is also restricted this can also lead to their speciation (Gyllenstrand et al., 2002; Pamilo & Rosengren, 1984; Seppä & Pamilo, 1995). A previous study that focused on a population of *E*. *ruidum* sp. 2 from Rosario Izapa in the state of Chiapas, southeast Mexico, showed that macrogynes and microgynes can be adopted by their monogynous and polygynous mother colonies, leading to low dispersal (Lenoir et al., 2011). Moreover, queen size dimorphism and social polymorphism has been observed in laboratory colonies both in *E*. *ruidum* sp. 3 and sp. 4 (K. I. Peña‐Carrillo, *unpublished data*), where few microgynes were produced. Further colony structure studies performed for species of the *E*. *ruidum* complex will reveal whether the presence of queen size dimorphism and social polymorphism was implicated in their diversification process.

*Ectatomma ruidum* has been the subject of a vast number of studies, including those on social structure (Corbara et al., 1989), foraging and diet (Lachaud, 1990; Riera‐Valera & Pérez‐Sánchez, 2009; Santamaría et al., 2009), macronutrient regulation (Cook & Behmer, 2010), home ranges and nestmate recognition (Breed et al., 1990), queen dimorphism (Lachaud et al., 1999; Lenoir et al., 2011), communication behavior (e.g., Pratt, 1989), and parasitoid interaction (Howard et al., 2001). Most of these studies, however, were based on specimens from different localities across the Neotropics. Our gathered evidence consistently shows that *E*. *ruidum* actually represents a species‐complex of species, which would change the interpretation and the extension of the conclusions that were drawn in previous studies. Our study thus highlights the importance of using different sources of molecular data for species delimitation of morphologically conserved taxa. Moreover, it is important to consider the occurrence of heteroplasmy in systematic studies, since it can lead to incorrect estimates of phylogenies if it is not detected. The presence of extensive heteroplasmy within the *E*. *ruidum* complex highlights the necessity of being aware of the occurrence of this phenomenon in other insect groups and metazoans in general, since it appears that it is not as rare as previously thought (Macey et al., 2021; Robison et al., 2015; White et al., 2008).

## CONFLICT OF INTEREST

The authors declare no conflict of interest.

## AUTHOR CONTRIBUTIONS

**Rubi N. Meza‐Lázaro:** Conceptualization (equal); Formal analysis (lead); Investigation (equal); Methodology (lead); Software (equal); Writing – original draft (lead); Writing – review & editing (equal). **Kenzy I. Peña‐Carrillo:** Data curation (equal); Formal analysis (supporting); Investigation (equal); Methodology (supporting); Writing – original draft (supporting); Writing – review & editing (supporting). **Chantal Poteaux:** Funding acquisition (supporting); Resources (equal); Validation (supporting); Writing – review & editing (equal). **Maria Cristina Lorenzi:** Resources (equal); Writing – review & editing (equal). **James Wetterer:** Resources (equal); Writing – review & editing (supporting). **Alejandro Zaldivar‐Riveron:** Conceptualization (equal); Funding acquisition (lead); Investigation (equal); Methodology (equal); Project administration (lead); Resources (equal); Supervision (lead); Writing – review & editing (lead).

## Supporting information

Fig S1

Fig S2

Table S1

Table S2

Table S3

Table S4

Table S5

Table S6

## Data Availability

3RAD and UCE raw reads generated in this study are deposited at the National Center for Biotechnology Sequence Read Archive (SRA; Bioproject ID PRJNA796376, PRJNA79660). All matrices that were analyzed in the study and their resulting topologies are available in the Figshare repository: https://doi.org/10.6084/m9.figshare.c.5821973.v1.

## References

[ece38704-bib-0001] Aberer, A. J. , Kobert, K. , & Stamatakis, A. (2014). Exabayes: Massively parallel bayesian tree inference for the whole‐genome era. Molecular Biology and Evolution, 31, 2553–2556. 10.1093/molbev/msu236 25135941 PMC4166930

[ece38704-bib-0002] Adams, S. A. , & Tsutsui, N. D. (2020). The evolution of species recognition labels in insects. Philosophical Transactions of the Royal Society B, 375, 20190476. 10.1098/rstb.2019.0476 PMC7331023 32420852

[ece38704-bib-0003] Aguilar‐Velasco, R. G. , Poteaux, C. , Meza‐Lázaro, R. , Lachaud, J.‐P. , Dubovikoff, D. , & Zaldívar‐Riverón, A. (2016). Uncovering species boundaries in the Neotropical ant complex *Ectatomma ruidum* (Ectatomminae) under the presence of nuclear mitochondrial paralogues. Zoological Journal of the Linnean Society, 178, 226–240. 10.1111/zoj.12407

[ece38704-bib-0004] Andermann, T. , Fernandes, A. M. , Olsson, U. , Töpel, M. , Pfeil, B. , Oxelman, B. , Aleixo, A. , Faircloth, B. C. , & Antonelli, A. (2019). Allele phasing greatly improves the phylogenetic utility of ultraconserved elements. Systematic Biology, 68, 32–46. 10.1093/sysbio/syy039 29771371 PMC6292485

[ece38704-bib-0005] Anderson, M. J. (2017). Permutational multivariate analysis of variance (PERMANOVA). Wiley StatsRef: Statistics Reference Online, 1–15. 10.1002/9781118445112.stat07841

[ece38704-bib-0006] Angers, B. , Leung, C. , Vétil, R. , Deremiens, L. , & Vergilino, R. (2018). The effects of allospecific mitochondrial genome on the fitness of northern redbelly dace (*Chrosomus eos*). Ecology and Evolution, 8, 3311–3321. 10.1002/ece3.3922 29607026 PMC5869299

[ece38704-bib-0007] Antweb. Version 8.64.2 . California Academy of Science. https://www.antweb.org

[ece38704-bib-0009] Bayona‐Vásquez, N. J. , Glenn, T. C. , Kieran, T. J. , Pierson, T. W. , Hoffberg, S. L. , Scott, P. A. , Bentley, K. E. , Finger, J. W. , Louha, S. , Troendle, N. , Diaz‐Jaimes, P. , Mauricio, R. , & Faircloth, B. C. (2019). Adapterama III: Quadruple‐indexed, double/triple‐enzyme RADseq libraries (2RAD/3RAD). PeerJ, 2019, 1–25. 10.7717/peerj.7724 PMC6791345 31616583

[ece38704-bib-0140] Blair, C. , Bryson, R. W. Jr , Linkem, C. W. , Lazcano, D. , Klicka, J. , & McCormack, J. E. (2018). Cryptic diversity in the Mexican highlands: Thousands of UCE loci help illuminate phylogenetic relationships, species limits and divergence times of montane rattlesnakes (Viperidae: Crotalus). Molecular Ecology Resources, 19, 349–365. 10.1111/1755-0998.12970 30565862

[ece38704-bib-0010] Blier, P. U. , Dufresne, F. , & Burton, R. S. (2001). Natural selection and the evolution of mtDNA‐encoded peptides: evidence for intergenomic co‐adaptation. TRENDS in Genetics, 17, 400–406. 10.1016/s0168-9525(01)02338-1 11418221

[ece38704-bib-0011] Blomquist, G. J. , & Bagnères, A. G. (2010). Insect hydrocarbons: Biology, biochemistry, and chemical ecology. Cambridge University Press.

[ece38704-bib-0012] Boomsma, J. J. , & Nash, D. R. (2014). Evolution: Sympatric speciation the eusocial way. Current Biology, 24, R798–R800. 10.1016/j.cub.2014.07.072 25202870

[ece38704-bib-0013] Bouckaert, R. , Heled, J. , Kühnert, D. , Vaughan, T. , Wu, C. H. , Xie, D. , Suchard, M. A. , Rambaut, A. , & Drummond, A. J. (2014). BEAST 2: A software platform for Bayesian evolutionary analysis. PLoS Computational Biology, 10, 1–6. 10.1371/journal.pcbi.1003537 PMC3985171 24722319

[ece38704-bib-0014] Bradbury, J. W. , & Vehrencamp, S. L. (1998). Principles of animal communication. Sinauer Associates Inc.

[ece38704-bib-0015] Branstetter, M. G. , Danforth, B. N. , Pitts, J. P. , Faircloth, B. C. , Ward, P. S. , Buffington, M. L. , Gates, M. W. , Kula, R. R. , & Brady, S. G. (2017). Phylogenomic insights into the evolution of stinging wasps and the origins of ants and bees. Current Biology, 27, 1019–1025. 10.1016/j.cub.2017.03.027 28376325

[ece38704-bib-0016] Breed, M. D. , Abel, P. , Bleuze, T. J. , & Denton, S. E. (1990). Thievery, home ranges, and nestmate recognition in *Ectatomma ruidum* . Oecologia, 84, 117–121. 10.1007/BF00665604 28312784

[ece38704-bib-0017] Brodetzki, T. R. , Inbar, S. , Cohen, P. , Aron, S. , Privman, E. , & Hefetz, A. (2019). the Interplay between Incipient species and social polymorphism in the desert ant cataglyphis. Scientific Reports, 9, 1–14. 10.1038/s41598-019-45950-1 31263177 PMC6603034

[ece38704-bib-0018] Brown, K. S. Jr (1981). The biology of Heliconius and related genera. Annual Review of Entomology, 26, 427–457. 10.1146/annurev.en.26.010181.002235

[ece38704-bib-0019] Bryant, D. , Bouckaert, R. , Felsenstein, J. , Rosenberg, N. A. , & Roychoudhury, A. (2012). Inferring species trees directly from biallelic genetic markers: Bypassing gene trees in a full coalescent analysis. Molecular Biology and Evolution, 29, 1917–1932. 10.1093/molbev/mss086 22422763 PMC3408069

[ece38704-bib-0020] Bryant, D. , & Moulton, V. (2004). Neighbor‐net: an agglomerative method for the construction of phylogenetic networks. Molecular Biology and Evolution, 21, 255–265. 10.1093/molbev/msh018 14660700

[ece38704-bib-0021] Burton, R. S. , & Barreto, F. S. (2012). A disproportionate role for mtDNA in Dobzhansky‐Muller incompatibilities? Molecular Ecology, 21, 4942–4957. 10.1111/mec.12006 22994153

[ece38704-bib-0022] Burton, R. S. , Pereira, R. J. , & Barreto, F. S. (2013). Cytonuclear genomic interactions and hybrid breakdown. Annual Review of Ecology, Evolution, and Systematics, 44, 281–302. 10.1146/annurev-ecolsys-110512-135758

[ece38704-bib-0023] Castresana, J. (2000). Selection of conserved blocks from multiple alignments for their use in phylogenetic analysis. Molecular Biology and Evolution, 17, 540–552. 10.1093/oxfordjournals.molbev.a026334 10742046

[ece38704-bib-0024] Catchen, J. M. , Amores, A. , Hohenlohe, P. , Cresko, W. , & Postlethwait, J. H. (2011). Stacks: building and genotyping loci de novo from short‐read sequences. G3: Genes|genomes|genetics, 1, 171–182. 10.1534/g3.111.000240 22384329 PMC3276136

[ece38704-bib-0025] Catchen, J. , Hohenlohe, P. A. , Bassham, S. , Amores, A. , & Cresko, W. A. (2013). Stacks: An analysis tool set for population genomics. Molecular Ecology, 22, 3124–3140. 10.1111/mec.12354 23701397 PMC3936987

[ece38704-bib-0026] Chifman, J. , & Kubatko, L. (2014). Quartet inference from SNP data under the coalescent model. Bioinformatics, 30, 3317–3324. 10.1093/bioinformatics/btu530 25104814 PMC4296144

[ece38704-bib-0027] Chou, J. Y. , & Leu, J. Y. (2010). Speciation through cytonuclear incompatibility: Insights from yeast and implications for higher eukaryotes. BioEssays, 32, 401–411. 10.1002/bies.200900162 20414898

[ece38704-bib-0028] Chou, J. Y. , & Leu, J. Y. (2015). The Red Queen in mitochondria: Cyto‐nuclear co‐evolution, hybrid breakdown and human disease. Frontiers in Genetics, 6, 1–8. 10.3389/fgene.2015.00187 26042149 PMC4437034

[ece38704-bib-0029] Chung, H. , & Carroll, S. B. (2015). Wax, sex and the origin of species: Dual roles of insect cuticular hydrocarbons in adaptation and mating. BioEssays, 37, 822–830. 10.1002/bies.201500014 25988392 PMC4683673

[ece38704-bib-0030] Clement, M. , Snell, Q. , Walker, P. , Posada, D. , & Crandall, K. (2002). TCS: Estimating gene genealogies. Parallel and Distributed Processing Symposium, International Proceedings. 2: 184. https://doi.org/10.1109/ipds.2002.1016585

[ece38704-bib-0031] Cook, S. C. , & Behmer, S. T. (2010). Macronutrient regulation in the tropical terrestrial ant *Ectatomma ruidum* (Formicidae): A field study in Costa Rica. Biotropica, 42, 135–139. 10.1111/j.1744-7429.2009.00616.x

[ece38704-bib-0032] Corbara, B. , Lachaud, J.‐P. , & Fresneau, D. (1989). Individual variability, social structure and division of labour in the ponerine ant *Ectatomma ruidum* Roger (Hymenoptera, Formicidae). Ethology, 82, 89–100. 10.1111/j.1439-0310.1989.tb00490.x

[ece38704-bib-0033] Coyne, J. A. , & Orr, H. A. (2004). Speciation. Sinauer Associates.

[ece38704-bib-0034] Crespi, B. , & Nosil, P. (2013). Conflictual speciation: Species formation via genomic conflict. Trends in Ecology and Evolution, 28, 48–57. 10.1016/j.tree.2012.08.015 22995895

[ece38704-bib-0035] Dayrat, B. (2005). Towards integrative taxonomy. Biological Journal of the Linnean Society, 85, 407–417. 10.1111/j.1095-8312.2005.00503.x

[ece38704-bib-0036] De Queiroz, K. (2007). Species concepts and species delimitation. Systematic Biology, 56, 879–886. 10.1080/10635150701701083 18027281

[ece38704-bib-0037] Driscoe, A. L. , Nice, C. C. , Busbee, R. W. , Hood, G. R. , Egan, S. P. , & Ott, J. R. (2019). Host plant associations and geography iteract to shape diversification in a specialist insect herbivore. Molecular Ecology, 28, 4197–4211. 10.1111/mec.15220 31478268

[ece38704-bib-0038] Dronnet, S. , Lohou, C. , Christides, J.‐P. , & Bagnères, A.‐G. (2006). Cuticular hydrocarbon composition reflects genetic relationship among colonies of the introduced termite *Reticulitermes santonensis* Feytaud. Journal of Chemical Ecology, 32, 1027–1042. 10.1007/s10886-006-9043-x 16739021

[ece38704-bib-0039] Eaton, D. A. R. (2014). PyRAD: Assembly of de novo RADseq loci for phylogenetic analyses. Bioinformatics, 30, 1844–1849. 10.1093/bioinformatics/btu121 24603985

[ece38704-bib-0040] Eaton, D. A. R. , & Overcast, I. (2020). Ipyrad: Interactive assembly and analysis of RADseq datasets. Bioinformatics, 36, 2592–2594. 10.1093/bioinformatics/btz966 31904816

[ece38704-bib-0041] Eaton, D. A. , & Ree, R. H. (2013). Inferring phylogeny and introgression using RADseq data: an example from flowering plants (Pedicularis: Orobanchaceae). Systematic Biology, 62, 689–706. 10.1093/sysbio/syt032 23652346 PMC3739883

[ece38704-bib-0042] Edgar, R. C. (2004). MUSCLE: multiple sequence alignment with high accuracy and high throughput. Nucleic Acids Research, 32, 1792–1797. 10.1093/nar/gkh340 15034147 PMC390337

[ece38704-bib-0043] Edwards, D. L. , & Knowles, L. L. (2014). Species detection and individual assignment in species delimitation: can integrative data increase efficacy? Proceedings of the Royal Society B: Biological Sciences, 281(1777), 20132765. 10.1098/rspb.2013.2765 PMC3896021 24403337

[ece38704-bib-0044] Emery, C. (1901). Notes sur les sous‐familles des Dorylines et Ponérines (Famille des Formicides). Annales De La Société Entomologique De Belgique, 45, 32–54.

[ece38704-bib-0045] Estrada, C. , & Jiggins, C. D. (2002). Patterns of pollen feeding and habitat preference among Heliconius species. Ecological Entomology, 27, 448–456. 10.1046/j.1365-2311.2002.00434.x

[ece38704-bib-0046] Evanno, G. , Regnaut, S. , & Goudet, J. (2005). Detecting the number of clusters of individuals using the software STRUCTURE: a simulation study. Molecular Ecology, 14(8), 2611–2620. 10.1111/j.1365-294X.2005.02553.x 15969739

[ece38704-bib-0047] Faircloth, B. C. (2013). Illumiprocessor: A trimmomatic wrapper for parallel adapter and quality trimming. 10.6079/J9ILL

[ece38704-bib-0048] Faircloth, B. C. (2016). PHYLUCE is a software package for the analysis of conserved genomic loci. Bioinformatics, 32, 786–788. 10.1093/bioinformatics/btv646 26530724

[ece38704-bib-0049] Faircloth, B. C. (2017). Identifying conserved genomic elements and designing universal bait sets to enrich them. Methods in Ecology and Evolution, 8, 1103–1112. 10.1111/2041-210X.12754

[ece38704-bib-0050] Faircloth, B. C. (2021). Tutorial II: Phasing UCE data. https://phyluce.readthedocs.io/en/latest/tutorials/tutorial‐2.html

[ece38704-bib-0051] Faircloth, B. C. , McCormack, J. E. , Crawford, N. G. , Harvey, M. G. , Brumfield, R. T. , & Glenn, T. C. (2012). Ultraconserved elements anchor thousands of genetic markers spanning multiple evolutionary timescales. Systematic Biology, 61, 717–726. 10.1093/sysbio/sys004 22232343

[ece38704-bib-0052] Feder, J. L. , Flaxman, S. M. , Egan, S. P. , & Nosil, P. (2013). Hybridization and the build‐up of genomic divergence during speciation. Journal of Evolutionary Biology, 26, 261–266. 10.1111/jeb.12009 23324002

[ece38704-bib-0053] Flouri, T. , Jiao, X. , Rannala, B. , & Yang, Z. (2018). Species tree inference with BPP using genomic sequences and the multispecies coalescent. Molecular Biology and Evolution, 35, 2585–2593. 10.1093/molbev/msy147 30053098 PMC6188564

[ece38704-bib-0054] Gershoni, M. , Templeton, A. R. , & Mishmar, D. (2009). Mitochondrial bioenergetics as a major motive force of speciation. BioEssays, 31, 642–650. 10.1002/bies.200800139 19408245

[ece38704-bib-0055] Glenn, T. C. , Nilsen, R. A. , Kieran, T. J. , Sanders, J. G. , Bayona‐Vásquez, N. J. , Finger, J. W. , Pierson, T. W. , Bentley, K. E. , Hoffberg, S. L. , & Louha, S. (2019). Adapterama I: universal stubs and primers for 384 unique dual‐indexed or 147,456 combinatorially‐indexed Illumina libraries (iTru \& iNext). PeerJ, 7, e7755. 10.7717/peerj.7755 31616586 PMC6791352

[ece38704-bib-0056] Grummer, J. A. , Bryson, R. W. , & Reeder, T. W. (2014). Species delimitation using bayes factors: Simulations and application to the *Sceloporus scalaris* species group (Squamata: Phrynosomatidae). Systematic Biology, 63, 119–133. 10.1093/sysbio/syt069 24262383

[ece38704-bib-0057] Grun, P. (1976). Cytoplasmic genetics and evolution. Columbia University Press.

[ece38704-bib-0058] Gyllenstrand, N. , Gertsch, P. J. , & Pamilo, P. (2002). Polymorphic microsatellite DNA markers in the ant *Formica exsecta* . Molecular Ecology Notes, 2, 67–69. 10.1046/j.1471-8286.2002.00152.x

[ece38704-bib-0059] Hakala, S. M. , Perttu, S. , & Helanterä, H. (2019). Evolution of dispersal in ants (Hymenoptera: Formicidae): A review on the dispersal strategies of sessile superorganisms. Myrmecological News, 29, 35–55. 10.25849/myrmecol.news_029:035

[ece38704-bib-0060] Harrison, R. G. , & Larson, E. L. (2014). Hybridization, introgression, and the nature of species boundaries. Journal of Heredity, 105, 795–809. 10.1093/jhered/esu033 25149255

[ece38704-bib-0061] Hartke, J. , Sprenger, P. P. , Sahm, J. , Winterberg, H. , Orivel, J. , Baur, H. , Beuerle, T. , Schmitt, T. , Feldmeyer, B. , & Menzel, F. (2019). Cuticular hydrocarbons as potential mediators of cryptic species divergence in a mutualistic ant association. Ecology and Evolution, 9, 9160–9176. 10.1002/ece3.5464 31463013 PMC6706187

[ece38704-bib-0062] Hebert, P. D. N. , Cywinska, A. , Ball, S. L. , & Dewaard, J. R. (2003). Biological identifications through DNA barcodes. Proceedings of the Royal Society of London. Series B: Biological Sciences, 270, 313–321. 10.1098/rspb.2002.2218 12614582 PMC1691236

[ece38704-bib-0063] Hernández‐Hernández, T. , Miller, E. C. , Román‐Palacios, C. , & Wiens, J. J. (2021). Speciation across the tree of life. Biological Reviews, 4, 1025–1242. 10.1111/brv.12698 33768723

[ece38704-bib-0064] Hill, G. E. (2015). Mitonuclear ecology. Molecular Biology and Evolution, 32, 1917–1927. 10.1093/molbev/msv104 25931514 PMC4833085

[ece38704-bib-0065] Hill, G. E. (2016). Mitonuclear coevolution as the genesis of speciation and the mitochondrial DNA barcode gap. Ecology and Evolution, 6, 5831–5842. 10.1002/ece3.2338 27547358 PMC4983595

[ece38704-bib-0066] Hill, G. E. (2017). The mitonuclear compatibility species concept. The Auk: Ornithological Advances, 134, 393–409. 10.1642/AUK-16-201.1

[ece38704-bib-0067] Hill, G. E. (2018). Mitonuclear mate choice: A missing component of sexual selection theory? BioEssays, 40, 1–10. 10.1002/bies.201700191 29405334

[ece38704-bib-0068] Hill, G. E. (2019). Reconciling the mitonuclear compatibility species concept with rampant mitochondrial introgression. Integrative and Comparative Biology, 59, 912–924. 10.1093/icb/icz019 30937430

[ece38704-bib-0069] Hochberg, M. E. , Sinervo, B. , & Brown, S. P. (2003). Socially mediated speciation. Evolution, 57, 154–158. 10.1111/j.0014-3820.2003.tb00224.x 12643576

[ece38704-bib-0070] Howard, R. W. , Pérez‐Lachaud, G. , & Lachaud, J.‐P. (2001). Cuticular hydrocarbons of *Kapala sulcifacies* (Hymenoptera: Eucharitidae) and its host, the ponerine ant *Ectatomma ruidum* (Hymenoptera: Formicidae). Annals of the Entomological Society of America, 94, 707–716.

[ece38704-bib-0071] Huson, D. H. , & Bryant, D. (2006). Application of phylogenetic networks in evolutionary studies. Molecular Biology and Evolution, 23, 254–267. 10.1093/molbev/msj030 16221896

[ece38704-bib-0072] Ilut, D. C. , Nydam, M. L. , & Hare, M. P. (2014). Defining loci in restriction‐based reduced representation genomic data from nonmodel species: sources of bias and diagnostics for optimal clustering. BioMed Research International, 2014, 1–9. 10.1155/2014/675158 PMC4095725 25057498

[ece38704-bib-0073] Johnstone, R. A. , Cant, M. A. , & Field, J. (2012). Sex‐biased dispersal, haplodiploidy and the evolution of helping in social insects. Proceedings of the Royal Society B: Biological Sciences, 279, 787–793. 10.1098/rspb.2011.1257 PMC3248733 21795270

[ece38704-bib-0074] Kass, R. E. , & Raftery, A. E. (1995). Bayes factors. Journal of the American Statistical Association, 90, 773–795. 10.1080/01621459.1995.10476572

[ece38704-bib-0075] Katoh, K. , Rozewicki, J. , & Yamada, K. D. (2019). MAFFT online service: multiple sequence alignment, interactive sequence choice and visualization. Briefings in Bioinformatics, 20, 1160–1166. 10.1093/bib/bbx108 28968734 PMC6781576

[ece38704-bib-0076] Kugler, C. , & Brown, W. L. (1982). Revisionary and other studies on the ant genus *Ectatomma*, including the descriptions of two new species. Estudios de revisión y de otros tipos en el género de hormigas *Ectatomma*, incluyendo las descripciones de dos nuevas especies. Agriculture, 24, 1–7.

[ece38704-bib-0077] Kulmuni, J. , & Westram, A. M. (2017). Intrinsic incompatibilities as a by‐product of divergent ecological selection: considering them in empirical studies on divergence with gene flow. Molecular Ecology, 26, 3093–3103. 10.1111/mec.14147 28423210

[ece38704-bib-0078] Kumar, S. , Stecher, G. , Li, M. , Knyaz, C. , & Tamura, K. (2018). MEGA X: Molecular evolutionary genetics analysis across computing platforms. Molecular Biology and Evolution, 35, 1547–1549. 10.1093/molbev/msy096 29722887 PMC5967553

[ece38704-bib-0079] Lachaud, J.‐P. (1990). Foraging activity and diet in some neotropical ponerine ants. I. *Ectatomma ruidum* Roger (Hymenoptera, Formicidae). Foliae Entomologica Mexicana, 78, 241–256.

[ece38704-bib-0080] Lachaud, J. P. , Cadena, A. , Schatz, B. , Pérez‐Lachaud, G. , & Ibarra‐Núñez, G. (1999). Queen dimorphism and reproductive capacity in the ponerine ant, Ectatomma ruidum Roger. Oecologia, 120, 515–523. 10.1007/s004420050885 28308301

[ece38704-bib-0081] Lanfear, R. , Frandsen, P. B. , Wright, A. M. , Senfeld, T. , & Calcott, B. (2017). Partitionfinder 2: New methods for selecting partitioned models of evolution for molecular and morphological phylogenetic analyses. Molecular Biology and Evolution, 34, 772–773. 10.1093/molbev/msw260 28013191

[ece38704-bib-0082] Leaché, A. D. , & Bouckaert, R. R. (2018). Species trees and species delimitation with SNAPP: A tutorial and worked example. Workshop on Population and Speciation Genomics, Český Krumlov. http://evomics.org/wp‐content/uploads/2018/01/BFD‐tutorial.pdf

[ece38704-bib-0083] Leaché, A. D. , & Fujita, M. K. (2010). Bayesian species delimitation in West African forest geckos (Hemidactylus fasciatus). Proceedings of the Royal Society B: Biological Sciences, 277, 3071–3077. 10.1098/rspb.2010.0662 PMC2982061 20519219

[ece38704-bib-0084] Leaché, A. D. , Fujita, M. K. , Minin, V. N. , & Bouckaert, R. R. (2014). Species delimitation using genome‐wide SNP Data. Systematic Biology, 63, 534–542. 10.1093/sysbio/syu018 24627183 PMC4072903

[ece38704-bib-0085] Leavitt, S. D. , Moreau, C. S. , & Lumbsch, H. T. (2015). The dynamic discipline of species delimitation: Progress toward effectively recognizing species boundaries in natural populations. In D. Upreti , P. Divakar , V. Shukla , & R. Bajpai (Eds.), Recent advances in lichenology (pp. 11–44). Springer. 10.1007/978-81-322-2235-4_2

[ece38704-bib-0086] Leigh, J. W. , & Bryant, D. (2015). POPART: Full‐feature software for haplotype network construction. Methods in Ecology and Evolution, 6, 1110–1116. 10.1111/2041-210X.12410

[ece38704-bib-0087] Lenoir, J. C. , Lachaud, J. P. , Nettel, A. , Fresneau, D. , & Poteaux, C. (2011). The role of the microgynes in the reproductive strategy of the neotropical ant *Ectatomma ruidum* . Naturwissenschaften, 98, 347–356. 10.1007/s00114-011-0774-3 21380620

[ece38704-bib-0088] Leonhardt, S. D. , Rasmussen, C. , & Schmitt, T. (2013). Genes versus environment: Geography and phylogenetic relationships shape the chemical profiles of stingless bees on a global scale. Proceedings of the Royal Society B: Biological Sciences, 280, 7–9. 10.1098/rspb.2013.0680 PMC3673053 23658202

[ece38704-bib-0089] Lucas, C. , Fresneau, D. , Kolmer, K. , Heinze, J. , Delabie, J. H. C. , & Pho, D. B. (2002). A multidisciplinary approach to discriminating different taxa in the species complex *Pachycondyla villosa* (Formicidae). Biological Journal of the Linnean Society, 75, 249–259. 10.1046/j.1095-8312.2002.00017.x

[ece38704-bib-0090] Macey, J. R. , Pabinger, S. , Barbieri, C. G. , Buring, E. S. , Gonzalez, V. L. , Mulcahy, D. G. , DeMeo, D. P. , Urban, L. , Hime, P. M. , Prost, S. , Elliot, A. N. , & Gemmel, N. J. (2021). Evidence of two deeply divergent co‐existing mitochondrial genomes in the Tuatara reveals an extremely complex genomic organization. Communications Biology, 4, 116. 10.1038/s42003-020-01639-0 33514857 PMC7846811

[ece38704-bib-0091] Mallet, J. , Beltrán, M. , Neukirchen, W. , & Linares, M. (2007). Natural hybridization in heliconiine butterflies: the species boundary as a continuum. BMC Evolutionary Biology, 7, 1–16. 10.1186/1471-2148-7-28 17319954 PMC1821009

[ece38704-bib-0092] Matute, D. R. , & Sepúlveda, V. E. (2019). Fungal species boundaries in the geomics era. Fungal Genetics and Biology, 131, 103249. 10.1016/j.fgb.2019.103249 31279976 PMC7355355

[ece38704-bib-0093] Meza‐Lázaro, R. N. , Poteaux, C. , Bayona‐Vásquez, N. J. , Branstetter, M. G. , & Zaldívar‐Riverón, A. (2018). Extensive mitochondrial heteroplasmy in the neotropical ants of the *Ectatomma ruidum* complex (Formicidae: Ectatomminae). Mitochondrial DNA Part A, 29, 1203–1214. 10.1080/24701394.2018.1431228 29385929

[ece38704-bib-0094] Miller, M. A. , Pfeiffer, W. , & Schwartz, T. (2010). Creating the CIPRES Science Gateway for inference of large phylogenetic trees. *2010 Gateway Computing Environments Workshop, GCE 2010*. 10.1109/GCE.2010.5676129

[ece38704-bib-0095] Mirarab, S. , Reaz, R. , Bayzid, M. S. , Zimmermann, T. , Swenson, S. , & Warnow, T. (2014). ASTRAL: Genome‐scale coalescent‐based species tree estimation. Bioinformatics, 30, 541–548. 10.1093/bioinformatics/btu462 PMC4147915 25161245

[ece38704-bib-0096] Mirarab, S. , & Warnow, T. (2015). ASTRAL‐II: Coalescent‐based species tree estimation with many hundreds of taxa and thousands of genes. Bioinformatics, 31, i44–i52. 10.1093/bioinformatics/btv234 26072508 PMC4765870

[ece38704-bib-0097] Mullen, S. P. , & Shaw, K. L. (2014). Insect speciation rules: unifying concepts in speciation research. Annual Review of Entomology, 59, 339–361. 10.1146/annurev-ento-120710-100621 24160421

[ece38704-bib-0098] Oksanen, J. , Blanchet, F. G. , Kindt, R. , Legendre, P. , Minchin, P. R. , Ohara, R. B. , Simpson, G. L. , Solymos, P. , Stevens, M. H. H. , & Wagner, H. (2016). Vegan: Community Ecology Package. R Package Version. 2.0‐10. https://github.com/vegandevs/vegan

[ece38704-bib-0099] Padial, J. M. , Miralles, A. , la Riva, I. , & Vences, M. (2010). The integrative future of taxonomy. Frontiers in Zoology, 7, 1–14. 10.1186/1742-9994-7-16 20500846 PMC2890416

[ece38704-bib-0100] Pamilo, P. , & Rosengren, R. (1984). Evolution of nesting strategies of ants: genetic evidence from different population types of *Formica* ants. Biological Journal of the Linnean Society, 21, 331–348. 10.1111/j.1095-8312.1984.tb00370.x

[ece38704-bib-0101] Pante, E. , Schoelinck, C. , & Puillandre, N. (2015). From integrative taxonomy to species description: One step beyond. Systematic Biology, 64, 152–160. 10.1093/sysbio/syu083 25358968

[ece38704-bib-0102] Pattengale, N. D. , Alipour, M. , Bininda‐Emonds, O. R. P. , Moret, B. M. E. , & Stamatakis, A. (2010). How many bootstrap replicates are necessary? Journal of Computational Biology, 17, 337–354. 10.1089/cmb.2009.0179 20377449

[ece38704-bib-0103] Peña‐Carrillo, K. I. , Lorenzi, M. C. , Brault, M. , Devienne, P. , Lachaud, J. P. , Pavan, G. , & Poteaux, C. (2021). A new putative species in the *Ectatomma ruidum* complex (Formicidae: Ectatomminae) produces a species‐specific distress call. Bioacoustics, 1–16. 10.1080/09524622.2021.1938226

[ece38704-bib-0104] Peña‐Carrillo, K. I. , Poteaux, C. , Leroy, C. , Meza‐Lázaro, R. N. , Lachaud, J. P. , Zaldívar‐Riverón, A. , & Lorenzi, M. C. (2021). Highly divergent cuticular hydrocarbon profiles in the clepotobiotic ants of the *Ectatomma ruidum* species complex. Chemoecology, 31, 125–135. 10.1007/s00049-020-00334-0

[ece38704-bib-0105] Pfennig, D. W. , Wund, M. A. , Snell‐Rood, E. C. , Cruickshank, T. , Schlichting, C. D. , & Moczek, A. P. (2010). Phenotypic plasticity’s impacts on diversification and speciation. Trends in Ecology and Evolution, 25, 459–467. 10.1016/j.tree.2010.05.006 20557976

[ece38704-bib-0106] Pratt, S. C. (1989). Recruitment and other communication behavior in the ponerine ant *Ectatomma ruidum* . Ethology, 81, 313–331. 10.1111/j.1439-0310.1989.tb00777.x

[ece38704-bib-0107] Presgraves, D. C. (2010). Speciation genetics: Search for the missing snowball. Current Biology, 20, R1073–R1074. 10.1016/j.cub.2010.10.056 21172625

[ece38704-bib-0108] Pritchard, J. K. , Stephens, M. , & Donnelly, P. (2000). Inference of population structure using multilocus genotype data. Genetics, 155, 945–959. 10.1093/genetics/155.2.945 10835412 PMC1461096

[ece38704-bib-0109] Quattrini, A. M. , Wu, T. , Soong, K. , Jeng, M. S. , Benayahu, Y. , & McFadden, C. S. (2019). A next generation approach to species delimitation reveals the role of hybridization in a cryptic species complex of corals. BMC Ecology and Evolution, 19, 116. 10.1186/s12862-019-1427-y PMC6555025 31170912

[ece38704-bib-0110] Rambaut, A. , Drummond, A. J. , Xie, D. , Baele, G. , & Suchard, M. A. (2018). Posterior summarization on Bayesian phylogenetics using Tracer 1.7. Systematic Biology, 67, 901–904. 10.1093/sysbio/syy032 29718447 PMC6101584

[ece38704-bib-0111] Riera‐Valera, M. A. , & Pérez‐Sánchez, A. J. (2009). Notas acerca de la dieta de *Ectatomma ruidum* (Roger 1861) (Hymenoptera: Formicidae: Ectatomminae) en un jardín venezolano. Boletín Sociedad Entomológica Aragonesa, 44, 550–552.

[ece38704-bib-0112] Robison, G. A. , Balvin, O. , Schal, C. , Vargo, E. L. , & Booth, W. (2015). Extensive mitochondrial heteroplasmy in natural populations of a resurging human pest, the bed bug (Hemiptera: Cimicidae). Journal of Medical Entomology, 52, 734–738. 10.1093/jme/tjv055 26335484 PMC4592348

[ece38704-bib-0113] Roger, J. (1860). Die Ponera‐artigen Ameisen. Berliner Entomologische Zeitschrift, 4, 278–312.

[ece38704-bib-0114] Rohland, N. , & Reich, D. (2012). Cost‐effective, high‐throughput DNA sequencing libraries for multiplexed target capture. Genome Research, 22, 939–946. 10.1101/gr.128124.111 22267522 PMC3337438

[ece38704-bib-0115] RStudio Team . (2021). RStudio: Integrated development ennvironment for R.RStudio, PBC. http://www.rstudio.com/

[ece38704-bib-0116] Santamaría, C. , Domínguez Haydar, Y. , & Armbrecht, I. (2009). Cambios en la distribución de nidos y abundancia de la hormiga *Ectatomma ruidum* (Roger 1861) en dos zonas de Colombia. Boletín Del Museo De Entomología De La Universidad Del Valle, 10, 10–18.

[ece38704-bib-0117] Savarit, F. , Sureau, G. , Cobb, M. , & Ferveur, J. F. (1999). Genetic elimination of known pheromones reveals the fundamental chemical bases of mating and isolation in Drosophila. Proceedings of the National Academy of Sciences of the United States of America, 96, 9015–9020. 10.1073/pnas.96.16.9015 10430887 PMC17724

[ece38704-bib-0118] Schlick‐Steiner, B. C. , Steiner, F. M. , Seifert, B. , Stauffer, C. , Christian, E. , & Crozier, R. H. (2010). Integrative taxonomy: a multisource approach to exploring biodiversity. Annual Review of Entomology, 55, 421–438. 10.1146/annurev-ento-112408-085432 19737081

[ece38704-bib-0119] Seehausen, O. , Butlin, R. K. , Keller, I. , Wagner, C. E. , Boughman, J. W. , Hohenlohe, P. A. , Peichel, C. L. , Saetre, G.‐P. , Bank, C. , Brännström, Å. , Brelsford, A. , Clarkson, C. S. , Eroukhmanoff, F. , Feder, J. L. , Fischer, M. C. , Foote, A. D. , Franchini, P. , Jiggins, C. D. , Jones, F. C. , … Widmer, A. (2014). Genomics and the origin of species. Nature Reviews Genetics, 15, 176–192. 10.1038/nrg3644 24535286

[ece38704-bib-0120] Seifert, B. (2010). Intranidal mating, gyne polymorphism, polygyny, and supercoloniality as factors for sympatric and parapatric speciation in ants. Ecological Entomology, 35, 33–40. 10.1111/j.1365-2311.2009.01136.x

[ece38704-bib-0121] Seo, T. K. (2008). Calculating bootstrap probabilities of phylogeny using multilocus sequence data. Molecular Biology and Evolution, 25, 960–971. 10.1093/molbev/msn043 18281270

[ece38704-bib-0122] Seppä, P. , & Pamilo, P. (1995). Gene flow and population viscosity in Myrmica ants. Heredity, 74(2), 200–209. 10.1038/hdy.1995.28

[ece38704-bib-0123] Simpson, J. T. , Wong, K. , Jackman, S. D. , Schein, J. E. , Jones, S. J. M. , & Birol, I. (2009). ABySS: a parallel assembler for short read sequence data. Genome Research, 19, 1117–1123. 10.1101/gr.089532.108 19251739 PMC2694472

[ece38704-bib-0124] Sloan, D. B. (2015). Using plants to elucidate the mechanisms of cytonuclear co‐evolution. New Phytologist, 205, 1040–1046. 10.1111/nph.12835 25729802

[ece38704-bib-0125] Smadja, C. , & Butlin, R. K. (2009). On the scent of speciation: The chemosensory system and its role in premating isolation. Heredity, 102, 77–97. 10.1038/hdy.2008.55 18685572

[ece38704-bib-0126] Smiley, J. (1978). Plant chemistry and the evolution of host specificity: New evidence from Heliconius and Passiflora. Science, 201, 745–747. 10.1126/science.201.4357.745 17750235

[ece38704-bib-0127] Snellings, Y. , Herrera, B. , Wildemann, B. , Beelen, M. , Zwarts, L. , Wenseleers, T. , & Callaerts, P. (2018). The role of cuticular hydrocarbons in mate recognition in Drosophila suzukii. Scientific Reports, 8, 1–11. 10.1038/s41598-018-23189-6 29567945 PMC5864920

[ece38704-bib-0128] Song, H. , Moulton, M. J. , & Whiting, M. F. (2014). Rampant nuclear insertion of mtDNA across diverse lineages with in Orthoptera (Insecta). PLoS One, 9, 41–43. 10.1371/journal.pone.0110508 PMC4204883 25333882

[ece38704-bib-0129] Sprenger, P. P. , & Menzel, F. (2020). Cuticular hydrocarbons in ants (Hymenoptera: Formicidae) and other insects: how and why they differ among individuals, colonies, and species. Myrmecological News, 30, 1–26. 10.25849/myrmecol.news_030:001

[ece38704-bib-0130] Stamatakis, A. (2014). RAxML version 8: A tool for phylogenetic analysis and post‐analysis of large phylogenies. Bioinformatics, 30, 1312–1313. 10.1093/bioinformatics/btu033 24451623 PMC3998144

[ece38704-bib-0131] Swofford, D. L. (2003). PAUP*. Phylogenetic Analysis Using Parsimony (*and Other Methods). Version 4. Sinauer Associates.

[ece38704-bib-0132] Talavera, G. , & Castresana, J. (2007). Improvement of phylogenies after removing divergent and ambiguously aligned blocks from protein sequence alignments. Systematic Biology, 56, 564–577. 10.1080/10635150701472164 17654362

[ece38704-bib-0133] van Zweden, J. S. , & d’Ettorre, P. (2010). Nestmate recognition in social insects and the role of hydrocarbons. In G. J. Blomquist & A.‐G. Bagnères (Eds.), Insect hydrocarbons: Biology, biochemistry and chemical ecology (pp. 222–243). Cambridge University Press.

[ece38704-bib-0134] White, D. J. , Wolff, J. N. , Pierson, M. , & Gemmell, N. J. (2008). Revealing the hidden complexities of mtDNA inheritance. Molecular Ecology, 17(23), 4925–4942. 10.1111/j.1365-294X.2008.03982.x 19120984

[ece38704-bib-0135] Will, K. W. , Mishler, B. D. , & Wheeler, Q. D. (2005). The perils of DNA barcoding and the need for integrative taxonomy. Systematic Biology, 54, 844–851. 10.1080/10635150500354878 16243769

[ece38704-bib-0136] Wollenberg Valero, K. C. , Marshall, J. W. , Bastiaans, E. , Caccone, A. , Camargo, A. , Morando, M. , Niemiller, M. L. , Pabijan, M. , Russello, M. A. , Sinervo, B. , Werneck, F. P. , Sites, J. W. , Wiens, J. J. , & Steinfartz, S. (2019). Patterns, mechanisms and genetics of speciation in reptiles and amphibians. Genes, 10, 646. 10.3390/genes10090646 PMC6769790 31455040

[ece38704-bib-0137] Yang, Z. , & Rannala, B. (2010). Bayesian species delimitation using multilocus sequence data. Proceedings of the National Academy of Sciences of the United States of America, 107, 9264–9269. 10.1073/pnas.0913022107 20439743 PMC2889046

[ece38704-bib-0138] Yang, Z. , & Rannala, B. (2014). Unguided species delimitation using DNA sequence data from multiple loci. Molecular Biology and Evolution, 31, 3125–3135. 10.1093/molbev/msu279 25274273 PMC4245825

[ece38704-bib-0139] Zaidi, A. A. , & Makova, K. D. (2019). Investigating mitonuclear interactions in human admixed populations. Nature Ecology and Evolution, 3, 213–222. 10.1038/s41559-018-0766-1 30643241 PMC6925600

