# Supplementary figures and images for: Genome and cuticular hydrocarbon‐based species delimitation shed light on potential drivers of speciation in a Neotropical ant species complex
Source: Ecol Evol. 2022 Mar 10;12(3):e8704. doi: 10.1002/ece3.8704 (PMC8928884; doi:10.1002/ece3.8704)

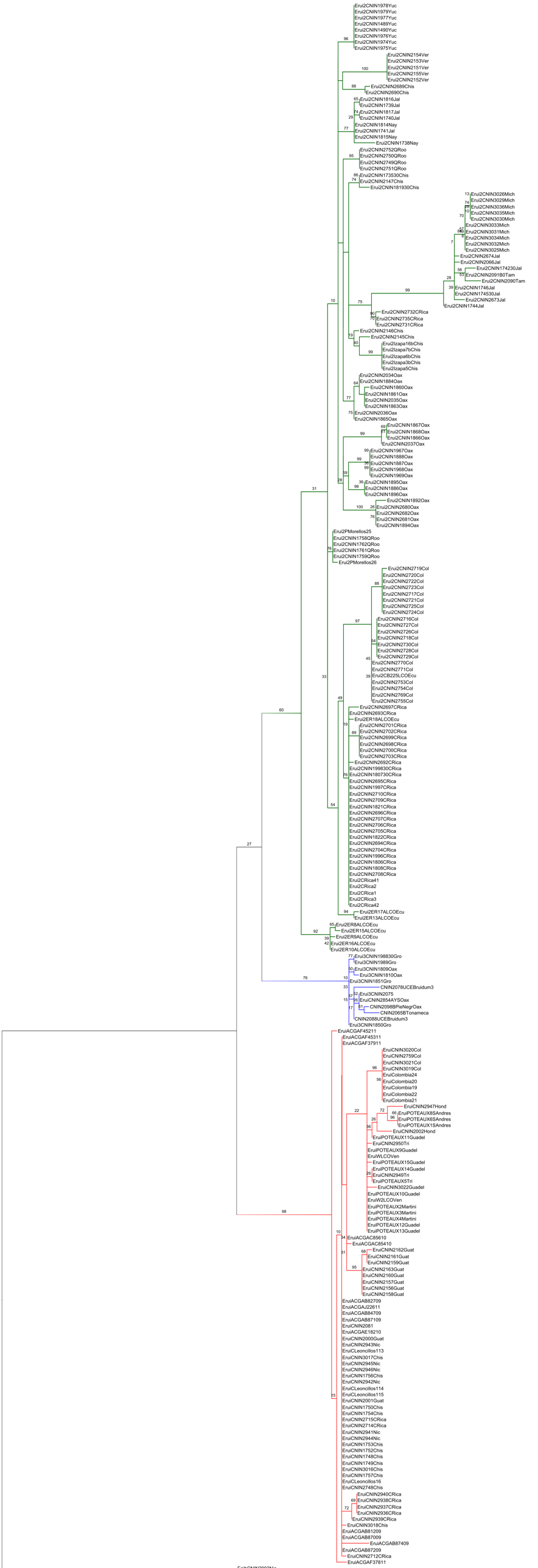

EgibCNIN2003Nic

Supplement: Supplementary file 1 — Fig S1 [file ECE3-12-e8704-s004.pdf]
