# Supplementary material for: Genome and cuticular hydrocarbon‐based species delimitation shed light on potential drivers of speciation in a Neotropical ant species complex
Source: Ecol Evol. 2022 Mar 10;12(3):e8704. doi: 10.1002/ece3.8704 (PMC8928884; doi:10.1002/ece3.8704)

A) 3RAD_ RAxML

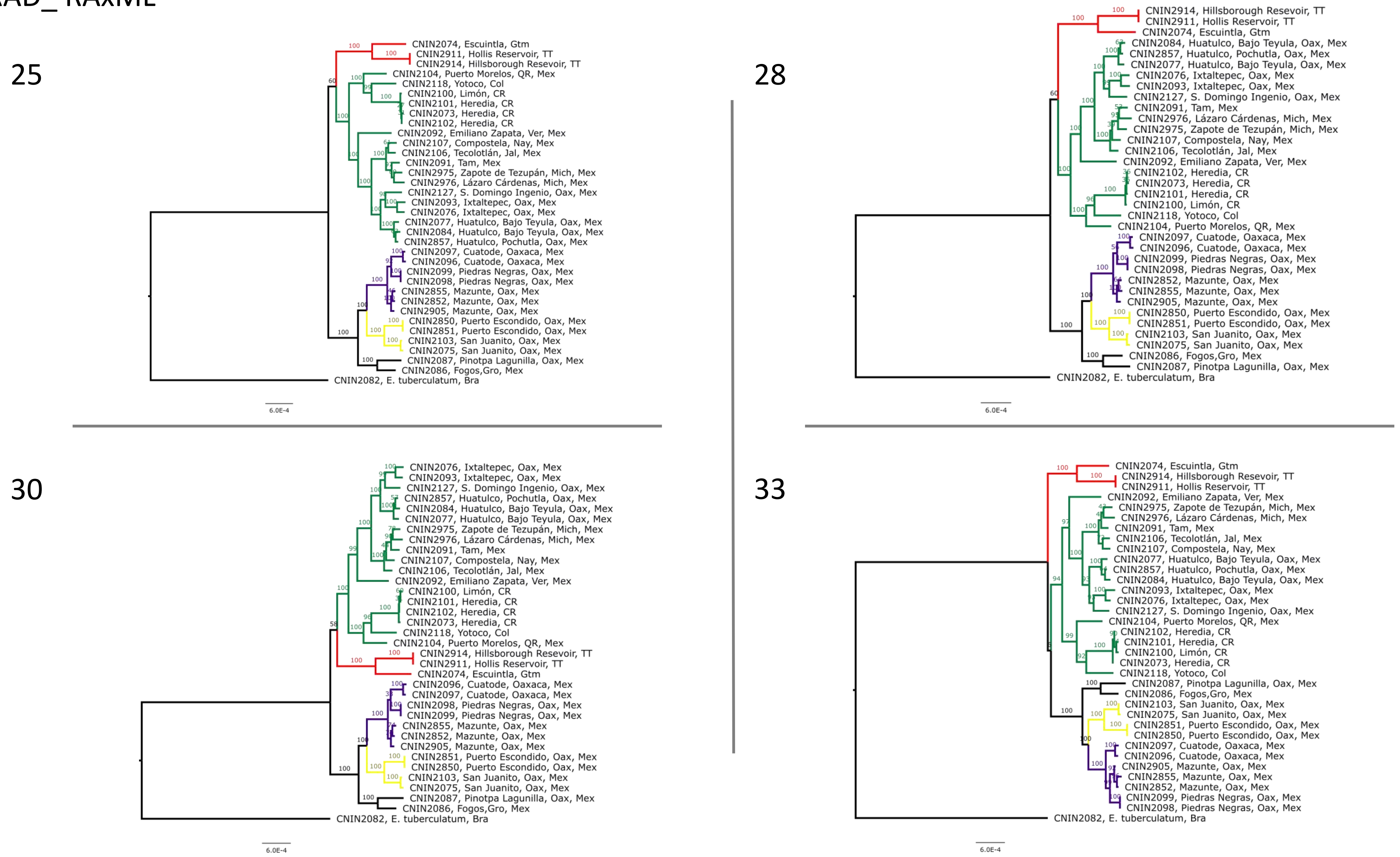
25
CNIN2074, Escuintla, Gtm
CNIN2911, Hollis Reservoir, TT
CNIN2914, Hillsborough Resevoir, TT
CNIN2104, Puerto Morelos, QR, Mex
CNIN2118, Yotoco, Col
CNIN2100, Limón, CR
CNIN2101, Heredia, CR
CNIN2073, Heredia, CR
CNIN2102, Heredia, CR
CNIN2092, Emiliano Zapata, Ver, Mex
CNIN2107, Compostela, Nay, Mex
CNIN2106, Tecolotlán, Jal, Mex
CNIN2091, Tam, Mex
CNIN2975, Zapote de Tezupán, Mich, Mex
CNIN2976, Lázaro Cárdenas, Mich, Mex
CNIN2127, S. Domingo Ingenio, Oax, Mex
CNIN2093, Ixtaltepec, Oax, Mex
CNIN2076, Ixtaltepec, Oax, Mex
CNIN2077, Huatulco, Bajo Teyula, Oax, Mex
CNIN2084, Huatulco, Bajo Teyula, Oax, Mex
CNIN2857, Huatulco, Pochutla, Oax, Mex
CNIN2097, Cuatode, Oaxaca, Mex
CNIN2096, Cuatode, Oaxaca, Mex
CNIN2099, Piedras Negras, Oax, Mex
CNIN2098, Piedras Negras, Oax, Mex
CNIN2855, Mazunte, Oax, Mex
CNIN2852, Mazunte, Oax, Mex
CNIN2905, Mazunte, Oax, Mex
CNIN2850, Puerto Escondido, Oax, Mex
CNIN2851, Puerto Escondido, Oax, Mex
CNIN2103, San Juanito, Oax, Mex
CNIN2075, San Juanito, Oax, Mex
CNIN2087, Pinotpa Lagunilla, Oax, Mex
CNIN2086, Fogos,Gro, Mex
CNIN2082, E. tuberculatum, Bra
6.0E-4
28
CNIN2914, Hillsborough Resevoir, TT
CNIN2911, Hollis Reservoir, TT
CNIN2074, Escuintla, Gtm
CNIN2084, Huatulco, Bajo Teyula, Oax, Mex
CNIN2857, Huatulco, Pochutla, Oax, Mex
CNIN2077, Huatulco, Bajo Teyula, Oax, Mex
CNIN2076, Ixtaltepec, Oax, Mex
CNIN2093, Ixtaltepec, Oax, Mex
CNIN2127, S. Domingo Ingenio, Oax, Mex
CNIN2091, Tam, Mex
CNIN2976, Lázaro Cárdenas, Mich, Mex
CNIN2975, Zapote de Tezupán, Mich, Mex
CNIN2107, Compostela, Nay, Mex
CNIN2106, Tecolotlán, Jal, Mex
CNIN2092, Emiliano Zapata, Ver, Mex
CNIN2102, Heredia, CR
CNIN2073, Heredia, CR
CNIN2101, Heredia, CR
CNIN2100, Limón, CR
CNIN2118, Yotoco, Col
CNIN2104, Puerto Morelos, QR, Mex
CNIN2097, Cuatode, Oaxaca, Mex
CNIN2096, Cuatode, Oaxaca, Mex
CNIN2099, Piedras Negras, Oax, Mex
CNIN2098, Piedras Negras, Oax, Mex
CNIN2852, Mazunte, Oax, Mex
CNIN2855, Mazunte, Oax, Mex
CNIN2905, Mazunte, Oax, Mex
CNIN2850, Puerto Escondido, Oax, Mex
CNIN2851, Puerto Escondido, Oax, Mex
CNIN2103, San Juanito, Oax, Mex
CNIN2075, San Juanito, Oax, Mex
CNIN2086, Fogos,Gro, Mex
CNIN2087, Pinotpa Lagunilla, Oax, Mex
CNIN2082, E. tuberculatum, Bra
6.0E-4
30
CNIN2076, Ixtaltepec, Oax, Mex
CNIN2093, Ixtaltepec, Oax, Mex
CNIN2127, S. Domingo Ingenio, Oax, Mex
CNIN2857, Huatulco, Pochutla, Oax, Mex
CNIN2084, Huatulco, Bajo Teyula, Oax, Mex
CNIN2077, Huatulco, Bajo Teyula, Oax, Mex
CNIN2975, Zapote de Tezupán, Mich, Mex
CNIN2976, Lázaro Cárdenas, Mich, Mex
CNIN2091, Tam, Mex
CNIN2107, Compostela, Nay, Mex
CNIN2106, Tecolotlán, Jal, Mex
CNIN2092, Emiliano Zapata, Ver, Mex
CNIN2100, Limón, CR
CNIN2101, Heredia, CR
CNIN2102, Heredia, CR
CNIN2073, Heredia, CR
CNIN2118, Yotoco, Col
CNIN2104, Puerto Morelos, QR, Mex
CNIN2914, Hillsborough Resevoir, TT
CNIN2911, Hollis Reservoir, TT
CNIN2074, Escuintla, Gtm
CNIN2096, Cuatode, Oaxaca, Mex
CNIN2097, Cuatode, Oaxaca, Mex
CNIN2098, Piedras Negras, Oax, Mex
CNIN2099, Piedras Negras, Oax, Mex
CNIN2855, Mazunte, Oax, Mex
CNIN2852, Mazunte, Oax, Mex
CNIN2905, Mazunte, Oax, Mex
CNIN2851, Puerto Escondido, Oax, Mex
CNIN2850, Puerto Escondido, Oax, Mex
CNIN2103, San Juanito, Oax, Mex
CNIN2075, San Juanito, Oax, Mex
CNIN2087, Pinotpa Lagunilla, Oax, Mex
CNIN2086, Fogos,Gro, Mex
CNIN2082, E. tuberculatum, Bra
6.0E-4
33
CNIN2074, Escuintla, Gtm
CNIN2914, Hillsborough Resevoir, TT
CNIN2911, Hollis Reservoir, TT
CNIN2092, Emiliano Zapata, Ver, Mex
CNIN2975, Zapote de Tezupán, Mich, Mex
CNIN2976, Lázaro Cárdenas, Mich, Mex
CNIN2091, Tam, Mex
CNIN2106, Tecolotlán, Jal, Mex
CNIN2107, Compostela, Nay, Mex
CNIN2077, Huatulco, Bajo Teyula, Oax, Mex
CNIN2857, Huatulco, Pochutla, Oax, Mex
CNIN2084, Huatulco, Bajo Teyula, Oax, Mex
CNIN2093, Ixtaltepec, Oax, Mex
CNIN2076, Ixtaltepec, Oax, Mex
CNIN2127, S. Domingo Ingenio, Oax, Mex
CNIN2104, Puerto Morelos, QR, Mex
CNIN2102, Heredia, CR
CNIN2101, Heredia, CR
CNIN2100, Limón, CR
CNIN2073, Heredia, CR
CNIN2118, Yotoco, Col
CNIN2087, Pinotpa Lagunilla, Oax, Mex
CNIN2086, Fogos,Gro, Mex
CNIN2103, San Juanito, Oax, Mex
CNIN2075, San Juanito, Oax, Mex
CNIN2851, Puerto Escondido, Oax, Mex
CNIN2850, Puerto Escondido, Oax, Mex
CNIN2097, Cuatode, Oaxaca, Mex
CNIN2096, Cuatode, Oaxaca, Mex
CNIN2905, Mazunte, Oax, Mex
CNIN2855, Mazunte, Oax, Mex
CNIN2852, Mazunte, Oax, Mex
CNIN2099, Piedras Negras, Oax, Mex
CNIN2098, Piedras Negras, Oax, Mex
CNIN2082, E. tuberculatum, Bra
6.0E-4

B) 3RAD_ExaBayes

25

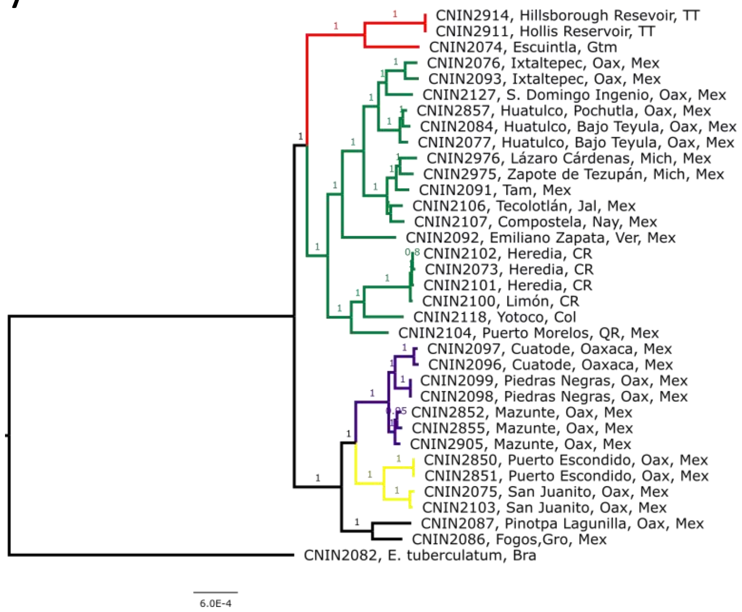

28

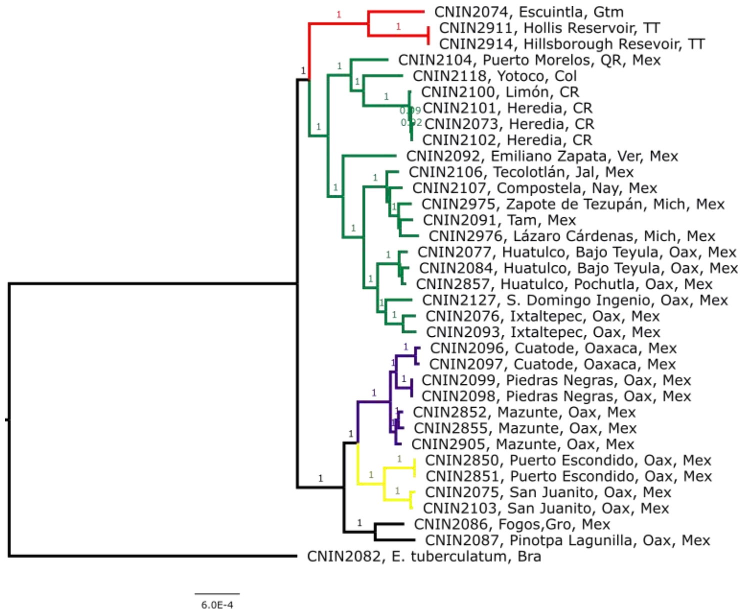

30

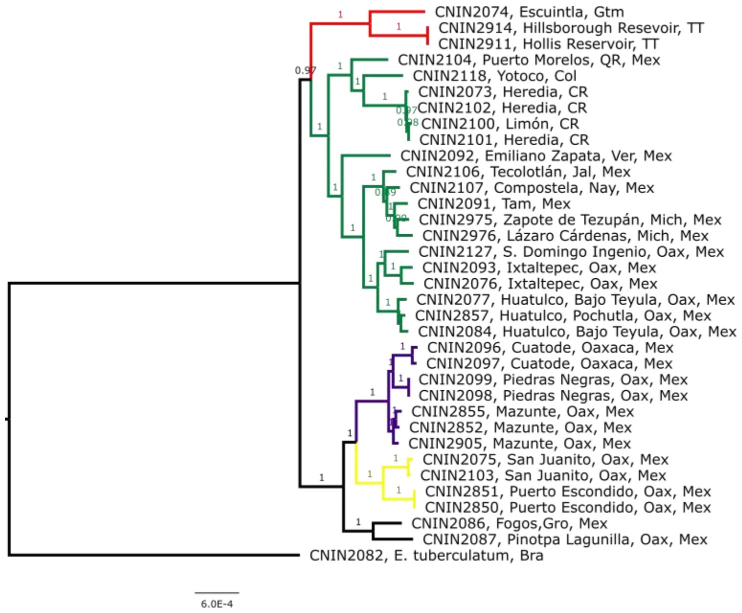

33

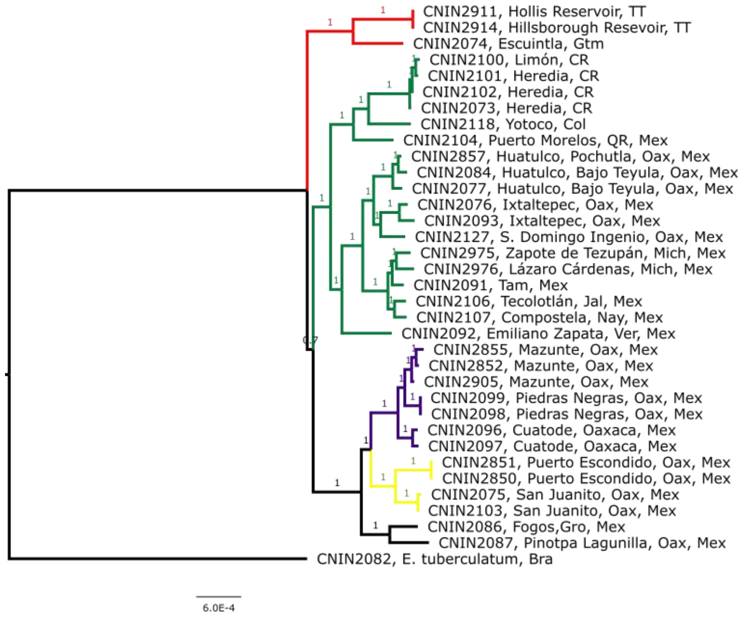

C) 3RAD_SVDquartets

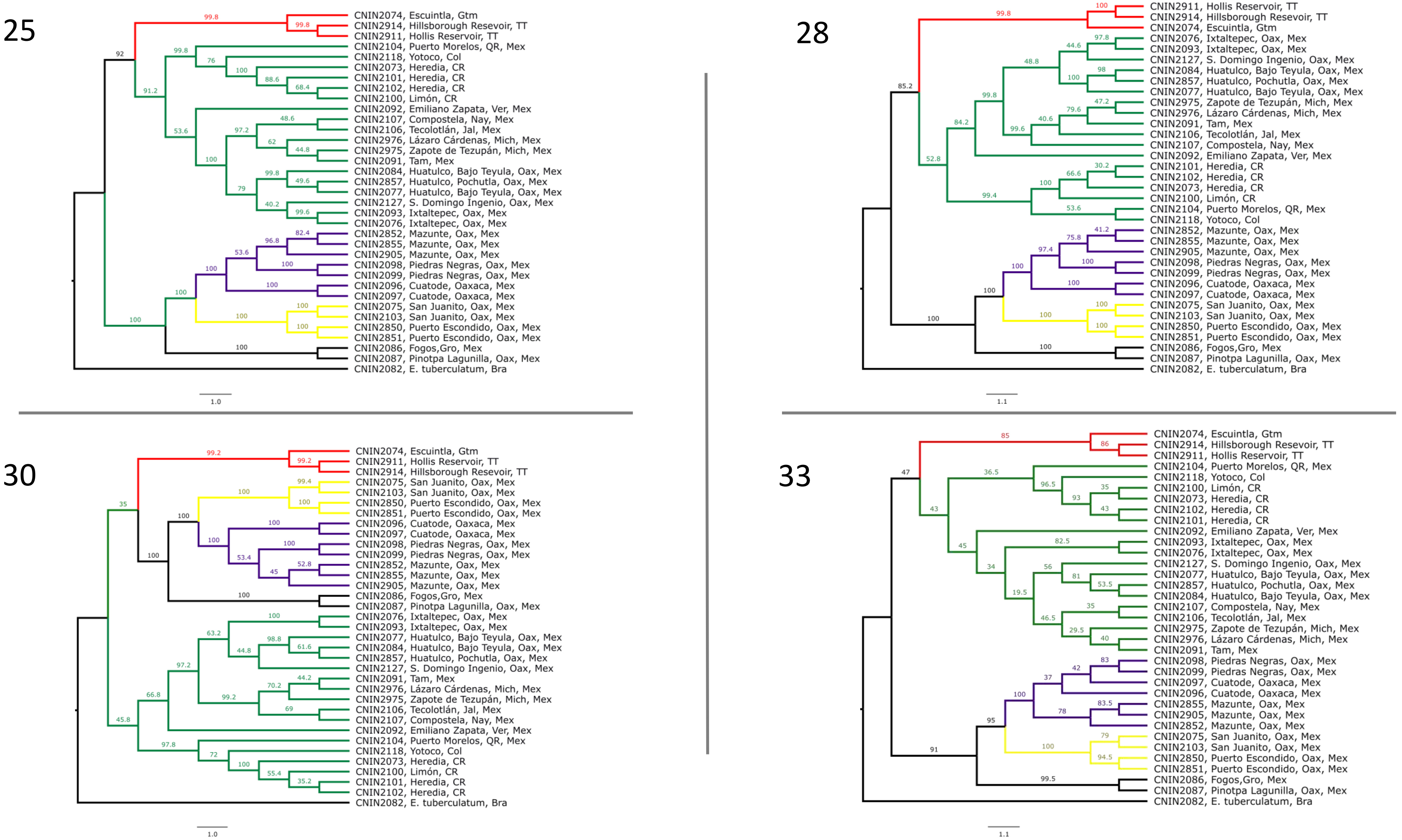

D) UCEs_RAxML

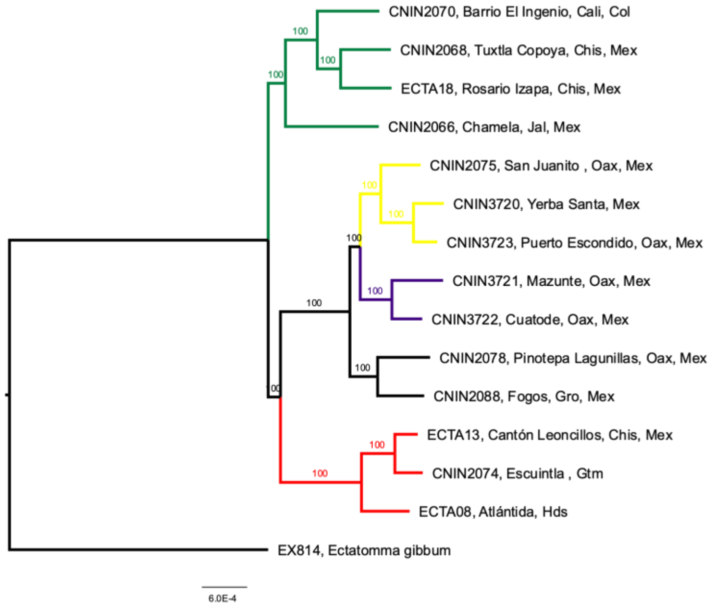

90%

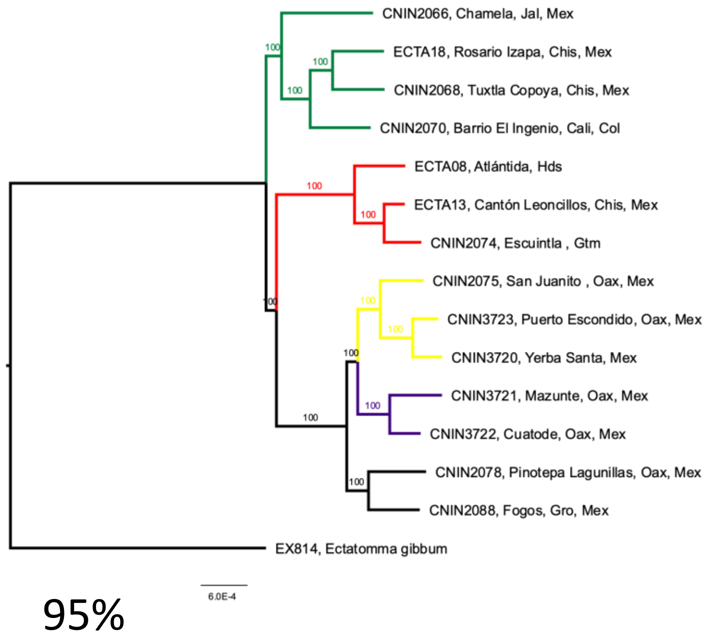

95%

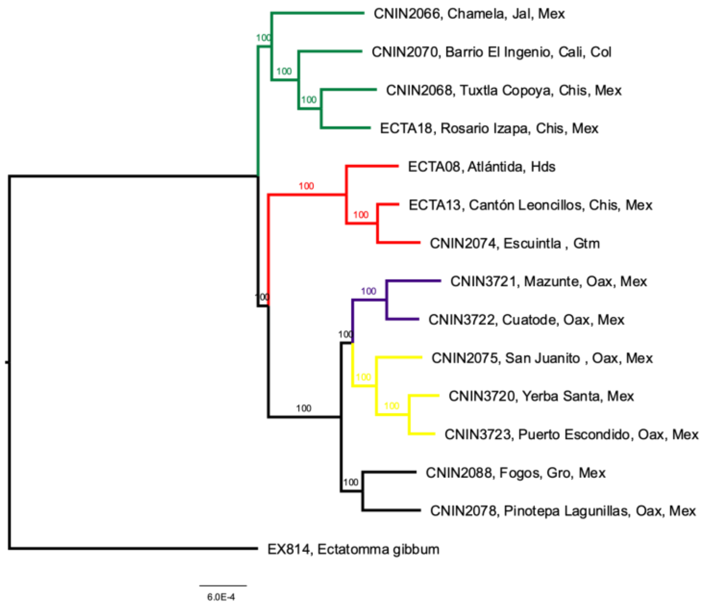

100%

E) UCEs_ExaBayes

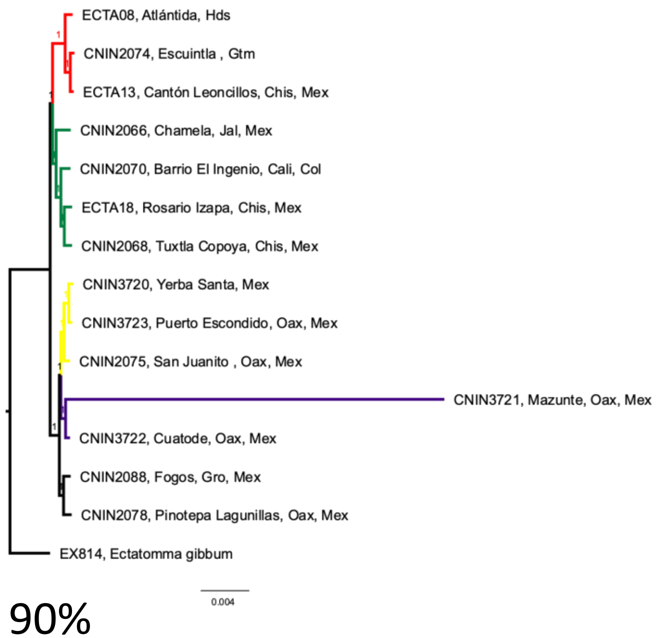

90%

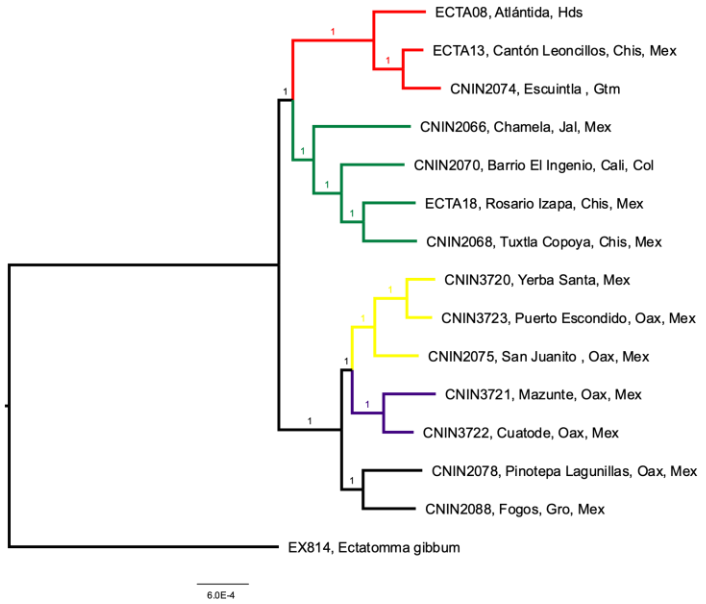

95%

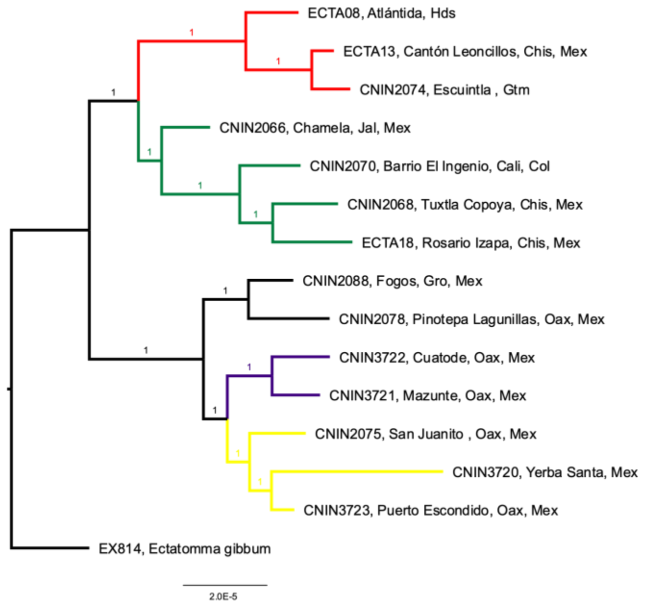

100%

F) UCEs_ ASTRAL_phased_90%

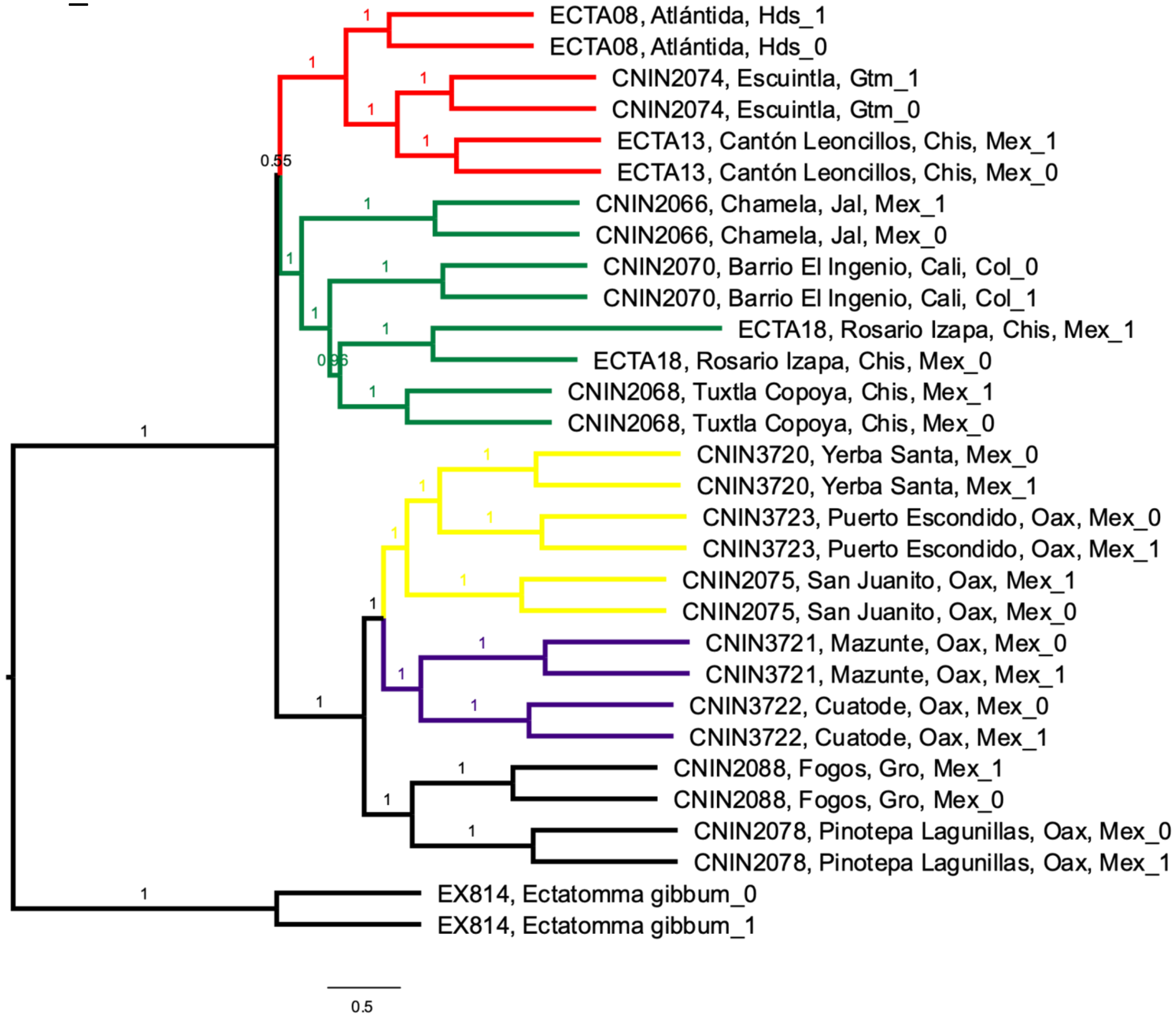

G) UCEs_SPLITSTREE_PHASED_90%

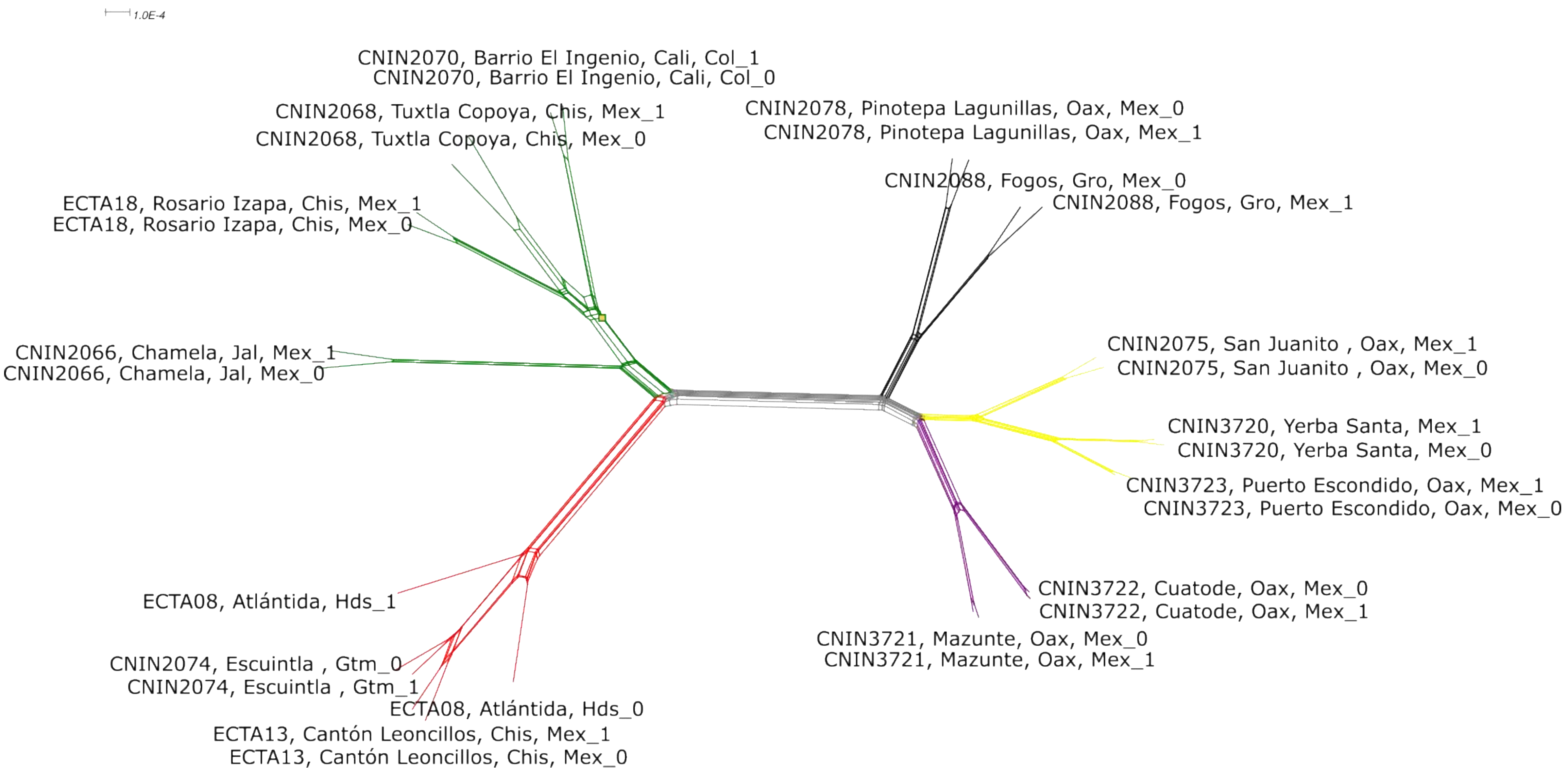

Supplement: Supplementary file 2 — Fig S2 [file ECE3-12-e8704-s001.pdf]
