# Supplementary material for: Genome and cuticular hydrocarbon‐based species delimitation shed light on potential drivers of speciation in a Neotropical ant species complex
Source: Ecol Evol. 2022 Mar 10;12(3):e8704. doi: 10.1002/ece3.8704 (PMC8928884; doi:10.1002/ece3.8704)
Supplement: Supplementary file 4 — Table S2 [file ECE3-12-e8704-s008.pdf]

Table S2. Statistics for different percentages of minimum taxon coverage for the 3RAD datasets. PIS= parsimony informative sites.

Data set	Clustering threshold	Minimum taxon coverage	Number of loci	Concatenated length (bp)	Missing data (%)	Number of Pis	Pis (%)
**With outgroup**							
**98_25**	0.98	25	7094	957769	15.59915313	8091	0.845
**98_28**	0.98	28	5437	734027	12.35893791	6172	0.840
**98_30**	0.98	30	3857	520660	9.644755186	4373	0.840
**98_33**	0.98	33	1154	155814	4.684129418	1318	0.846
**Without outgroup**							
**98_25**	0.98	25	7052	952059	13.062	8058	0.846
**98_28**	0.98	28	5361	723729	9.838	6091	0.842
**98_30**	0.98	30	3718	501860	7.067	4235	0.844
**98_33**	0.98	33	986	133135	2.094	1185	0.890
