# Supplementary material for: Genome and cuticular hydrocarbon‐based species delimitation shed light on potential drivers of speciation in a Neotropical ant species complex
Source: Ecol Evol. 2022 Mar 10;12(3):e8704. doi: 10.1002/ece3.8704 (PMC8928884; doi:10.1002/ece3.8704)
Supplement: Supplementary file 5 — Table S3 [file ECE3-12-e8704-s003.pdf]

Table S3. Statistics for different percentages of minimum taxon coverage for the UCEs datasets. PIS= parsimony informative sites.

Matrix completeness percentage	Loci	Characters	Variable	PIS	Singletons	Conserved
Unphased						
75%	2196	1817455	27939	8309	19630	1789561
80%	2046	1722246	26547	7931	18616	1695699
85%	2046	1722246	26547	7931	18616	1695699
90%	1267	1023170	16071	4967	11104	1007099
95%	827	650790	10398	3103	7295	640392
100%	642	508859	8228	2488	5740	500631
Phased						
85%	737	702500	12166	9841	2323	690272
95%	606	580450	10103	8165	1936	570304
100%	390	376393	6720	5407	1313	369631
