# Supplementary material for: Genome and cuticular hydrocarbon‐based species delimitation shed light on potential drivers of speciation in a Neotropical ant species complex
Source: Ecol Evol. 2022 Mar 10;12(3):e8704. doi: 10.1002/ece3.8704 (PMC8928884; doi:10.1002/ece3.8704)

Table S4. BPP Species delimitation based on 200 (A) and 50 (B) loci matrices with three and ten replicates (r) respectively. We included models with PP= posterior probabilities ≥ 0.1. The columns represent three sets of parameters varying the ancestral population size (θ) and root age (τ). Each number (1 or 0) represents one of the nodes, from 10 to 17, according to the guide tree (C). Zeros represent collapsed nodes.

**A**	θ G(1, 10) and τ0G(1, 10)	θ G(1, 10) and τ0G(2, 2000)	θ G(2, 200) and τ0G(2, 2000)	θ G(2, 2000) and τ0G(2, 2000)
No-data	**10000000**	**-**	**100000000**	**10000000 / 11000000**
	0.999	<0.1	0.943	0.626 / 0.312
r0	**11111111**	**11111111**	**11111111**	**11111111**
	0.902	1	1	1
r1	**11110011**	**11110011 / 11111011**	**11110011**	**11111111**
	0.979	0.555 / 0.445	0.9949	0.944
r2	**11110011**	**11110011**	**11111111**	**11111111**
	0.999	0.999	1	0.985

**B**	θ G(1, 10) and τ0G(1, 10)	θ G(1, 10) and τ0G(2, 2000)	θ G(2, 200) and τ0G(2, 2000)	θ G(2, 2000) and τ0G(2, 2000)
No-data	**10000011**	**-**	**-**	**11000011**
	0.264	<0.1	<0.1	0.197
r0	**11111111 / 11110011 / 11101111**	**11111111**	**11111111**	**11111111**
	0.505 / 0.234 / 0.214	0.923	0.944	1
r1	**11111111**	**11111111**	**11111111**	**11111111**
	0.906	0.990	0.989	1
r2	**11111111**	**11111111 / 11110011 / 11111011**	**11111111 / 11110011**	**11111111**
	0.908	0.555 / 0.312	0.498 / 0.433	0.999
r3	**11111111 / 11110011**	**11111111**	**11111111**	**11111111**
	0.524 / 0.461	1.0	1.0	1.0
r4	**11110011 / 11111011 / 11111111**	**11111111 / 11111011 / 11110011**	**11111111 / 11111011 / 11110011**	**11111111**
	0.672 / 0.150 / 0.160	0.471/ 0.423 / 0.105	0.59661 / 0.26722 / 0.13617	0.98741
r5	**11101111**	**11101111 / 11111111**	**11101111 / 11111111**	**11111111**
	0.911	0.614 / 0.386	0.709 / 0.291	0.977
r6	**11101111 / 11111111**	**11100011 / 11110011**	**11101111 / 11111111 / 11100011**	**11101111 / 11111111**
	0.531 / 0.262	0.646 / 0.263	0.461 / 0.213 / 137	0.526 / 0.473
r7	**11111111**	**11111111 / 11101111**	**11111111**	**11111111**

	0.956	0.949 / 0.048	0.999	1.0
r8	**11111111**	**11111111 / 11101111**	**11111111 / 11101111**	**11111111**
	0.976	0.737 / 0.263	0.764 / 0.236	1.0
r9	**11111111**	**11111111**	**11111111**	**11111111**
	1.0	0.999	0.993	0.999

C

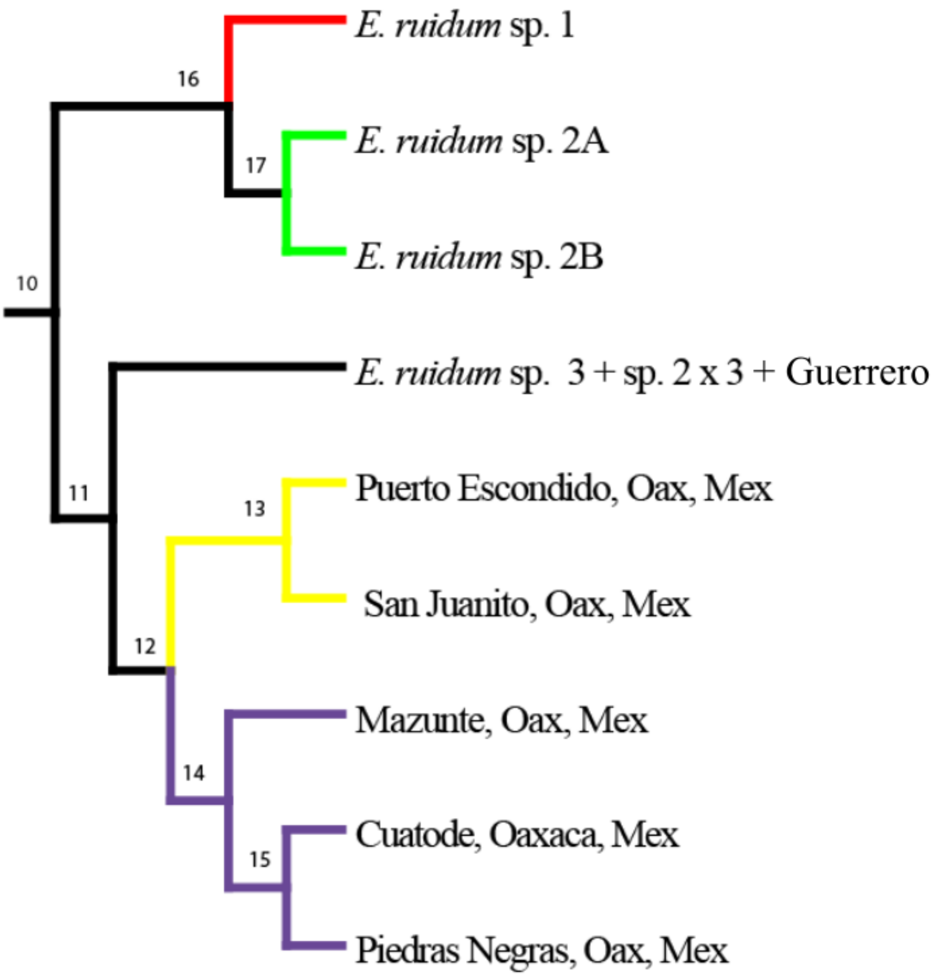

Supplement: Supplementary file 6 — Table S4 [file ECE3-12-e8704-s002.pdf]
