# Supplementary material for: Genome and cuticular hydrocarbon‐based species delimitation shed light on potential drivers of speciation in a Neotropical ant species complex
Source: Ecol Evol. 2022 Mar 10;12(3):e8704. doi: 10.1002/ece3.8704 (PMC8928884; doi:10.1002/ece3.8704)
Supplement: Supplementary file 7 — Table S5 [file ECE3-12-e8704-s005.pdf]

Table S5. Bayes factor comparisons of the two species delimitation models using the phased UCE dataset. MLE = Marginal likelihood estimate; BF = Bayes factor.

Model	MLE 24 steps	BF = 2 (MLE1 - MLE0)
**1 species**	-5210.9685	
**2 species** (*E. ruidum* sp. 1+ *E. ruidum* sp. 2, *E. ruidum* sp. 3 **+** *E. ruidum* sp. 4 + *E. ruidum* sp. 2x3 + Guerrero)	-4970.0676	481.802
**3 species** (*E. ruidum* sp. 1 + *E. ruidum* sp. 2, *E. ruidum* sp. 3 + *E. ruidum* sp. 4, *E. ruidum* sp. 2x3 + Guerrero)	-4676.8785	0.5372
**3 species B** (*E. ruidum* sp. 1, *E. ruidum* sp. 2, *E. ruidum* sp. 3 + *E. ruidum* sp. 4 + *E. ruidum* sp. 2x3 + Guerrero)	-4637.5183	78.7204
**4 species** (*E. ruidum* sp. 1, *E. ruidum* sp. 2, *E. ruidum* sp. 3+ *E. ruidum* sp. 4, *E. ruidum* sp. 2x3 + Guerrero)	-4405.8586	463.3194
**4 species B** (*E. ruidum* sp. 1+ *E. ruidum* sp. 2, *E. ruidum* sp. 3, *E. ruidum* sp. 4, *E. ruidum* sp. 2x3 + Guerrero)	-4677.1471	585.8409
**5 species** (*E. ruidum* sp. 1, *E. ruidum* sp. 2, *E. ruidum* sp. 3, *E. ruidum* sp. 4, *E. ruidum* sp. 2x3 + Guerrero)	-4318.5253	174.6666
MLE = Marginal likelihood estimate		
BF = Bayes factor		
