# Supplementary material for: Genome and cuticular hydrocarbon‐based species delimitation shed light on potential drivers of speciation in a Neotropical ant species complex
Source: Ecol Evol. 2022 Mar 10;12(3):e8704. doi: 10.1002/ece3.8704 (PMC8928884; doi:10.1002/ece3.8704)
Supplement: Supplementary file 8 — Table S6 [file ECE3-12-e8704-s006.pdf]

Supplementary Table S6. Chemical (A), genetic (B,C,D, E) and geographic (F,G,H) distances for the examined populations using the Mantel tests.

**A) Chemical distances (CHC)**								
	Tapachula	Cali	Coyula	Puerto Morelos	Puerto Escondido	Piedras Negras	Mazunte	Cuatode
Tapachula	-	-	-	-	-	-	-	-
Cali	36.36	-	-	-	-	-	-	-
Coyula	52.276	36.477	-	-	-	-	-	-
Puerto Morelos	36.425	23.223	35.708	-	-	-	-	-
Puerto Escondido	48.312	37.9	46.479	39.666	-	-	-	-
Piedras Negras	57.064	37.632	41.54	46.031	30.13	-	-	-
Mazunte	56.131	37.005	46.886	45.117	23.732	23.508	-	-
Cuatode	55.826	35.794	40.275	42.329	29.403	15.646	20.28	-

**B) 3RAD genetic distances**								
	sp1	Cali	Coyula	PuertoMorelos	PuertoEscondido	PiedrasNegras	Mazunte	Cuatode
sp1	-	-	-	-	-	-	-	-
Cali	0.1484	-	-	-	-	-	-	-
Coyula	0.1379	0.0953	-	-	-	-	-	-
PuertoMorelos	0.136	0.0618	0.0852	-	-	-	-	-
PuertoEscondido	0.1782	0.1614	0.1496	0.1495	-	-	-	-
PiedrasNegras	0.1717	0.1551	0.1413	0.1437	0.0827	-	-	-
Mazunte	0.1696	0.1508	0.1385	0.1395	0.0791	0.0164	-	-
Pcuatode	0.1684	0.1518	0.138	0.1406	0.0817	0.0277	0.022	-

**C) UCEs genetic distances**					
Genetic	Tapachula	Cali	PuertoCEscondido	Mazunte	Cuatode
Tapachula	-	-	-	-	-

Cali	0.00274	-	-	-	-
Puerto Escondido	0.00348	0.00318	-	-	-
Mazunte	0.00358	0.00323	0.00183	-	-
Cuatode	0.00337	0.00302	0.00174	0.00109	-

**D) COI primary haplotypes genetic distances**								
	Tapachula	Cali	Coyula	PuertoMorelos	PuertoEscondido	PiedrasNegras	Mazunte	Cuatode
Tapachula	-	-	-	-	-	-	-	-
Cali	0.05847	-	-	-	-	-	-	-
Coyula	0.05844	0.02168	-	-	-	-	-	-
PuertoMorelos	0.04952	0.01693	0.01413	-	-	-	-	-
PuertoEscondido	0.05864	0.05011	0.05269	0.04424	-	-	-	-
PiedrasNegras	0.06230	0.05378	0.05680	0.04792	0.00480	-	-	-
Mazunte	0.06230	0.05059	0.05680	0.04792	0.00800	0.00639	-	-
Cuatode	0.06070	0.05218	0.05516	0.04633	0.00320	0.00160	0.00479	-

**E) COI secondary haplotypes genetic distances**								
	Tapachula	Cali	Coyula	PuertoMorelos	PuertoEscondido	PiedrasNegras	Mazunte	Cuatode
Tapachula	-	-	-	-	-	-	-	-
Cali	0.0586	-	-	-	-	-	-	-
Coyula	0.0578	0.0214	-	-	-	-	-	-
PuertoMorelos	0.0560	0.0194	0.0138	-	-	-	-	-
PuertoEscondido	0.0672	0.0616	0.0667	0.0592	-	-	-	-
PiedrasNegras	0.0880	0.0760	0.0744	0.0736	0.0748	-	-	-
Mazunte	0.0608	0.0520	0.0545	0.0448	0.0416	0.0656	-	-
Cuatode	0.1024	0.0904	0.0853	0.0848	0.0896	0.0352	0.0800	-

F) Geographic distances (km) among populations for CHCs vs COI primary and secondary datasets								
	Tapachula	Cali	Coyula	Puerto Morelos	Puerto Escondido	Piedras Negras	Mazunte	Cuatode
Tapachula	-	-	-	-	-	-	-	-
Cali	2150	-	-	-	-	-	-	-
Coyula	430	2565	-	-	-	-	-	-
Puerto Morelos	892	2243	1140	-	-	-	-	-
Puerto Escondido	520	2645	85	1205	-	-	-	-
Piedras Negras	470	2595	40	1175	50	-	-	-
Mazunte	460	2585	30	1170	60	11	-	-
Cuatode	455	2580	23	1160	65	16	8	-

G) Geographic distances (km) among populations for 3RAD dataset								
	sp1	Cali	Coyula	Puerto Morelos	Puerto Escondido	Piedras Negras	Mazunte	Cuatode
sp1	-	-	-	-	-	-	-	-
Cali	2059	-	-	-	-	-	-	-
Coyula	639	2565	-	-	-	-	-	-
Puerto Morelos	799	2243	1140	-	-	-	-	-
Puerto Escondido	725	2645	85	1205	-	-	-	-
Piedras Negras	678	2595	40	1175	50	-	-	-
Mazunte	667	2585	30	1170	60	11	-	-
Cuatode	662	2580	23	1160	65	16	8	-

H) Geographic distances (km) among populations for UCEs dataset					
	Tapachula	Cali	Puerto Escondido	Mazunte	Cuatode
Tapachula	-	-	-	-	-
Cali	2150	-	-	-	-
PuertoEscondido	520	2645	-	-	-
Mazunte	460	2585	60	-	-
Cuatode	455	2580	65	8	-
